# Molecular classification of gastric cancer: Towards a pathway-driven targeted therapy

**DOI:** 10.18632/oncotarget.4990

**Published:** 2015-07-22

**Authors:** Ismael Riquelme, Kathleen Saavedra, Jaime A. Espinoza, Helga Weber, Patricia García, Bruno Nervi, Marcelo Garrido, Alejandro H. Corvalán, Juan Carlos Roa, Carolina Bizama

**Affiliations:** ^1^ Department of Pathology, School of Medicine, Universidad de La Frontera, CEGIN-BIOREN, Temuco, Chile; ^2^ Department of Pathology, School of Medicine, Pontificia Universidad Católica de Chile, Santiago, Chile; ^3^ UC-Center for Investigational Oncology (CITO), Pontificia Universidad Católica de Chile, Santiago, Chile; ^4^ Department of Hematology Oncology, School of Medicine, Pontificia Universidad Católica de Chile, Santiago, Chile; ^5^ Millennium Institute on Immunology and Immunotherapy, Pontificia Universidad Católica de Chile, Santiago, Chile; ^6^ Advanced Center for Chronic Diseases (ACCDIS), Pontificia Universidad Católica de Chile, Santiago, Chile

**Keywords:** gastric cancer, chemotherapy, molecular classification, signaling pathway, cancer stem cells

## Abstract

Gastric cancer (GC) is the third leading cause of cancer mortality worldwide. Although surgical resection is a potentially curative approach for localized cases of GC, most cases of GC are diagnosed in an advanced, non-curable stage and the response to traditional chemotherapy is limited. Fortunately, recent advances in our understanding of the molecular mechanisms that mediate GC hold great promise for the development of more effective treatment strategies. In this review, an overview of the morphological classification, current treatment approaches, and molecular alterations that have been characterized for GC are provided. In particular, the most recent molecular classification of GC and alterations identified in relevant signaling pathways, including ErbB, VEGF, PI3K/AKT/mTOR, and HGF/MET signaling pathways, are described, as well as inhibitors of these pathways. An overview of the completed and active clinical trials related to these signaling pathways are also summarized. Finally, insights regarding emerging stem cell pathways are described, and may provide additional novel markers for the development of therapeutic agents against GC. The development of more effective agents and the identification of biomarkers that can be used for the diagnosis, prognosis, and individualized therapy for GC patients, have the potential to improve the efficacy, safety, and cost-effectiveness for GC treatments.

## INTRODUCTION

Gastric cancer (GC) is an important public health problem worldwide due to its high incidence and mortality. Currently, GC is the fifth most frequently diagnosed cancer, and it is the third most lethal cancer worldwide. Each year, almost one million new cases of GC are diagnosed and more than 700,000 people die of this disease, thereby representing ~10% of the deaths due to cancer worldwide [1]. This high mortality rate is associated with the absence of significant symptoms in the early stages of GC, the lack of validated screening programs, and cancer health care in developing countries, which can be economically or geographically difficult to access. As a result, many cases of GC are diagnosed at an advanced stage and have a poor prognosis.

GC is a complex and multifactorial disease. There are many inherited and environmental factors that play a role in GC carcinogenesis, including the genetic characteristics of the host, infectious agents (such as *Helicobacter pylori* and Epstein Barr), and dietary habits [2]. Correspondingly, GC is a heterogeneous disease, both histologically and genetically, and patient outcome is difficult to predict using just the classical histologic and molecular classification criteria [3]. The current histologic classification of GC is well accepted and several molecular analyses have associated the genetic and epigenetic alterations with the prognosis and diagnosis of advanced stage patients. However, the prognosis and predictive capacity of this system do not adequately guide patient management, thereby necessitating the development of robust classifiers [4, 5]. Recent advances in high-throughput technologies, including microarrays and next-generation sequencing, have led to the discovery of new molecular markers, intracellular pathways, and molecular subtypes of GC. The resulting data have strengthened the rationale of current experimental therapies for various stages of clinical validation, and have elucidated novel treatment options that are currently under investigation. The overall aim is to improve the effectiveness of current therapeutic regimens and to improve patient quality of life.

Here, we review management strategies for cases of advanced stage GC, current knowledge regarding the molecular classification of GC, and we discuss the emerging role of signaling pathways that are affected in GC and that provide the identification of new therapeutic targets for this disease.

## MANAGEMENT OF ADVANCED STAGE GC

The outcome for patients with GC is predicted based on the initial stage of the disease at diagnosis. Localized disease that is limited to the mucosa and submucosa is frequently cured with surgical treatment. The five-year survival rate for these cases is 70-90% [6, 7]. However, upon invasion of the sub-mucosa by GC, the risk of lymph node metastases increases and patient survival decreases. Correspondingly, the five-year survival rate following radical gastrectomy without any further treatment is 10-30% [7, 8]. Several strategies have been developed to improve overall survival (OS) for cases involving locally advanced disease. The strategies that have achieved some survival benefit compared with surgery alone include adjuvant chemoradiotherapy (CRT), perioperative chemotherapy, and adjuvant chemotherapy.

The adjuvant CRT has been considered standard therapy in the USA since the publication of the phase III Intergroup-0116 (INT 0116). This study included 566 patients who received surgery alone or a CRT regimen of 5-fluorouracil (5-FU) plus leucovorin followed by 4500 cGy radiation. The median OS period for the surgery-only group was 27 months, compared with 36 months for the CRT group. In addition, the hazard ratio (HR) for death was 1.35 (95% confidence interval (CI): 1.09-1.66; *P* = 0.005), and the HR for relapse was 1.52 (95% CI: 1.23-1.86; *P* < 0.001) [9]. However, this study has been criticized for the limited lymph node dissections performed on the patients enrolled, the complexity of the CRT protocol and for the rate of serious toxicity. Furthermore, there was no evidence for the effectiveness of postoperative adjuvant CRT, and radiotherapy helped only in patients with resected gastric cancer with high-risk loco-regional failure [10]. In contrast to the USA experience, the standard surgery accompanied by D2 lymphadenectomy is performed routinely in Japan. The Korean phase III ARTIST study [11], included 458 GC patients that underwent D2 resection, and were randomly assigned to receive adjuvant capecitabine plus cisplatin (XP), or XP plus radiotherapy (XP/XRT/XP). Overall, the addition of CRT did not benefit GC patients in the chemotherapy alone group, with a 3-year disease-free survival (DFS) rate for the XP/XRT/XP group versus the XP group being 78.2% and 74.2%, respectively (*P* = 0.0862). However, in a subgroup of patients with lymph node-positive disease, the 3-year DFS rates were 77.5% for the XP/XRT/XP group and 72.3% for the XP alone group (*P* = 0.0365).

In the phase III MAGIC trial, 503 patients were enrolled and the benefit of an epirubicine/cisplatin/5-FU (ECF) regimen for perioperative chemotherapy versus surgery alone was demonstrated. The five-year survival rates were 36% versus 23%, respectively (HR = 0.75, 95% CI: 0.60-0.93, *P* = 0.009) and the former group had a higher progression-free survival (PFS) rate (HR = 0.66, 95% CI: 0.53-0.81, *P* < 0.001). Other potential benefits of this strategy included tumor downstaging, which increased the likelihood of curative resection, improved patient survival with the elimination of micrometastases, a rapid improvement in symptoms, and a determination of tumor sensitivity to chemotherapy [12].

In a meta-analysis conducted by the Global Advanced/Adjuvant *Stomach Tumor* Research International Collaboration (GASTRIC) group [13], 3,838 patients from 17 different trials were examined. A modest, yet statistically significant benefit was observed for the estimated median survival period following the use of post-operative chemotherapy versus surgery alone (7.8 years *vs*. 4.9 years, respectively; HR = 0.82, 95% CI: 0.76-0.90, *P* = 0.001). In the Asian group Adjuvant Chemotherapy Trial of TS-1 for Gastric Cancer (ACTS-GC), S-1, an oral fluoropyrimidine chemotherapy agent, was investigated [14]. In this randomized study of 1,059 patients with stage II-III GC, the 3-year OS rate was 80% versus 70% for surgery alone. Finally, in a capecitabine and oxaliplatin adjuvant study for stomach cancer (CLASSIC), a phase III randomized controlled trial involving 37 centers in South Korea, China, and Taiwan [15], patients with stage II-IIIB GC who underwent curative gastrectomy were randomly assigned to receive adjuvant chemotherapy capecitabine plus intravenous oxaliplatin or surgery alone. The 3-year DFS rates were 74% (95% CI: 69-79) and 59% (95% CI: 53-64), respectively (HR = 0.56, 95% CI: 0.44-0.72; *P* < 0.0001).

For cases involving advanced, incurable GC, several studies have shown the benefit of palliative chemotherapy. For example, in a meta-analysis of 35 clinical studies and a total of 5,726 patients that was performed to evaluate chemotherapy for cases of advanced stage GC in 2010, a significant benefit in OS was observed for the group that received chemotherapy versus best supportive care (HR = 0.37, 95% CI: 0.24-0.55) [16]. Overall, there has been little progress in the evaluation of new chemotherapy regimens, and the classical regimen of cisplatin plus 5-FU (FP4w) remains the reference regimen. However, there are drugs that have recently been added to third generation regimens, and these include capecitabine, docetaxel, S-1, oxaliplatin, and irinotecan. Pivotal phase III trials suggest that oral XP and S-1 can provide an effective first-line treatment and can replace infused 5-FU [17–19]. S-1 plus oxaliplatin (SOX) is regarded as a palliative standard first-line chemotherapy in Japan. A phase III study suggested that S-1 plus oxaliplatin (SOX) was non-inferior to cisplatin plus S-1 (CS) in terms of PFS and OS. However, SOX provided a considerable advantage in safety over CS [20]. The regimen FLO (5-FU, leucovorin, oxaliplatin) appeared to reduce the hematological and cardiovascular toxicity and may be an alternative to cisplatin regimen (FLP) for the treatment of advanced GC [21]. Furthermore, the combination of epirubicine, capecitabine, and oxaliplatin (EOX) has been found to be as effective as the ECF regimen [22]. In a phase III study of docetaxel [23, 24], and in a study of weekly administrations of paclitaxel and irinotecan [25, 26], the potential for these treatments to serve as second-line chemotherapy regimens were evaluated. Selected phase III clinical trials of current chemotherapy regimens for adjuvant and palliative chemotherapy for advanced stage GC are summarized in Table 1.

**Table 1:** Pivotal phase III trials using cytotoxic chemotherapy for GC

Phase III trial	Patients	Treatment	Clinical efficiency*				Condition
**Adjuvant chemotherapy**			**Follow-up**	**mRFS (%)**	**Survival rates (%)**	***P-value***	
**INT 0116 trial** **Mcdonald et al. (2001)[9]**	556	FL	3 years	31	41	0.005	Resectable GC or GEJ cancer
		FL/RT		48	50		
**ARTIST trial** **Lee et al. (2012) [11]**	458	XP	7 years	74.2	73	NS	Resectable GC with D2 lymph node dissection
		XP/XRT/XP		78.2	75		
**MAGIC trial** **Cunningham et al. (2006) [12]**	503	Surgery alone	5 years	18	26	0.009	Resectable GC or GEJ cancer
		Perioperative ECF + surgery		32	36.3		
**Asian ACTS-GC trial** **Sakuramoto et al (2007) [14]**	1059	Surgery alone	3 years	59.6	70.1	0.003	Resectable GC with D2 lymph node dissection
		Surgery + S-1		72.2	80.1		
**CLASSIC trial** **Noh et al. (2014) [15]**	1035	Surgery alone	5 years	53	69	0.0015	Resectable GC with D2 lymph node dissection
		XP		68	78		
**Palliative chemotherapy**			**ORR (%)**	**mRFS (months)**	**mOS (months)**	***P-value***	
**V325 trial** **Ajani et al. (2007) [24]**	445	DCF	37	5.6	9.2	0.02	Advanced stage GC and GEJ cancer
		FP4w	25	3.7	8.6		
**ML17032** **Kang et al. (2009) [18]**	316	XP	46	5.6	10.5	0.008	Advanced stage GC
		FP3w	32	5.0	9.3		
**SPIRITS trial** **Koizumi et al. (2008) [19]**	305	S-1P	54	6.0	13	0.04	Advanced stage GC
		S-1	31	4.0	11		
**Yamada et al. (2015) [20]**	685	S-1	52,2	5,4	13,1	NR	HR: 0.93 Non Inferior
		SOX	55,7	5,5	14,1		
**Al-Batran et al. (2008) [21]**	220	FLO	35	5.8	10.7	0.08	Metastatic GC and GEJ cancer
		FLP	25	3.9	8.8		
**REAL 2 trial** **Cunningham et al. (2008) [22]**	1002	ECF	41	6.2	9.9	0.06	Advanced stage GC and GEJ cancer
		ECX	46	6.7	9.9		
		EOF	42	6.5	9.3		
		EOX	48	7.0	11.2		
**COUGAR-02 trial** **Ford at al. (2014) [23]**	168	Docetaxel	53	3.1	5.2	0.01	Refractory treatment fluoropyrimidime plus platinum advanced GC
		Symptom control		2.0	3.6		
**French trial** **Dank et al. (2008) [25]**	333	IFL	33	5.0	9.0	NS	Advanced stage and metastatic GC
		FP4w	26	4.2	8.7		
**WJOG 4007 trial** **Hironaka et al (2013) [26]**	223	Paclitaxel	21	3.6	9.5	NS	Refractory treatment fluoropyrimidime plus platinum advanced stage GC
		Irinotecan	14	2.3	8.4		

mRFS: mean relapse-free survival; ORR: overall response rate; mPFS: mean progression-free survival; mOS: mean overall survival; FL: 5-fluorouracil (5-FU), leucovorin; RT: radiotherapy; XP: capecitabine, cisplatin; ECF: epirubicin, cisplatin, 5-FU; DCF: docetaxel, cisplatin, 5-FU; FP4w: 5-FU, cisplatin every 4 weeks; FP3w: 5-FU, cisplatin every 3 weeks; EOX: epirubicin, oxaliplatin, capecitabine; ECX: epirubicin, cisplatin, capecitabine; EOF: epirubicin, oxaliplatin, 5-FU; S-1P: S-1, cisplatin; SOX: S-1 plus oxaliplatin; FLO: 5-FU, leucovorin, oxaliplatin; FLP: 5-FU, leucovorin, cisplatin; IFL: Irinotecan, 5-FU, leucovorin.

* The clinical efficacy was calculated from published data.

The first targeted treatment for GC patients that was approved by the Federal Drug Administration (FDA) was trastuzumab in combination with chemotherapy (cisplatin plus either capecitabine or 5-FU). Currently, this treatment regimen is available in the clinic as a first-line therapy for patients with metastatic cancer of the stomach or gastroesophageal junction (GEJ) positive for ERBB2 protein (also called HER2). In the phase III ToGA trial, an increase in OS was observed for patients with ERBB2-positive GC or GEJ that were treated with chemotherapy plus trastuzumab compared to chemotherapy alone [27]. Patients that are diagnosed with metastatic stomach cancer must be evaluated for ERBB2 overexpression or amplification by immunohistochemistry or fluorescence *in situ* hybridization (FISH), respectively. Typically, only 10-30% of these patients are ERBB2-positive and are eligible for treatment with Herceptin plus chemotherapy [28–31]. Thus, an active area of research is the identification of new molecular therapeutic targets that provide more specific and effective treatments for disease. To understand the impact of using molecular targets for personalized medicine, and why they may be useful in the treatment of GC patients, it is important to describe the histogenetic sequence and the molecular classification of GC that was recently published [4].

## MULTISTEP CARCINOGENESIS

**Figure 1:**
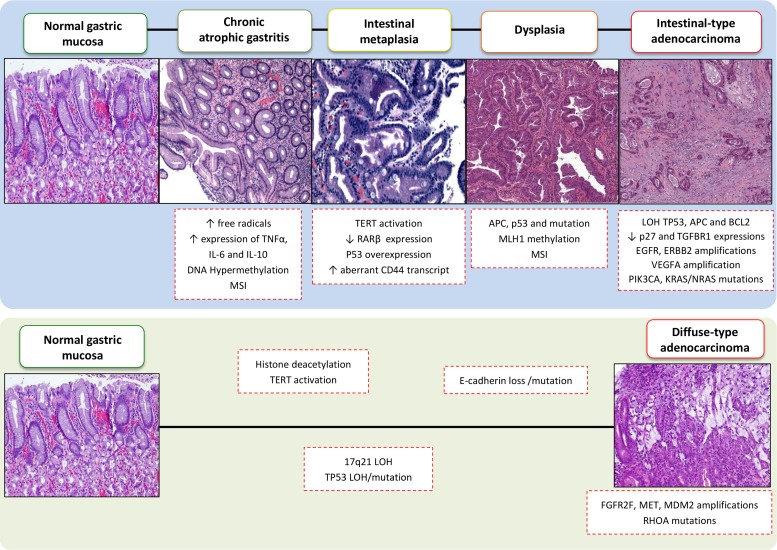
Sequential morphologic, genetic and epigenetic alterations in multistep gastric carcinogenesis This figure summarizes the sequence of molecular events that have been characterized for intestinal-type and diffuse-type GC according to the Correa cascade model. MSI: microsatellite instability; GS: genomically stable; EBV: Epstein-Barr virus; CIN: chromosomally unstable; LOH: loss of heterozygosity.

The majority of GC patients are diagnosed with adenocarcinoma histology (90%); while the remaining 10% of patients are diagnosed with lymphoma or gastrointestinal stromal tumors. In general, there are two types of gastric adenocarcinoma, intestinal-type (50%) and diffuse-type (33%), according to the Lauren classification [32]. The remaining 17% of gastric adenocarcinomas are classified as mixed or are unclassified. Intestinal-type adenocarcinoma is most frequently diagnosed, and it is preceded by the development of gastric lesions known as Correa's cascade [33] (Figure 1). These lesions have been associated with *H. pylori* infection that possess *cag* pathogenicity island and secrete a functional cytotoxin more severe gastric injury and further augment the risk for developing dismal gastric cancer [34]. In contrast, diffuse-type adenocarcinoma has a poorer prognosis, it generally occurs in younger patients, and it can occur anywhere in the stomach, yet it especially affects the cardia [35].

In 1992, Correa proposed that gastric carcinogenesis is a multistep process that involves a sequence of histological changes that lead to intestinal-type adenocarcinoma. These steps include the development of chronic gastritis, atrophy, intestinal metaplasia, and eventually dysplasia that results in GC (Figure 1). Recent molecular studies have provided evidence to support Correa's model. For example, the initial steps of stomach carcinogenesis have been characterized by genetic instability, telomerase activation, *TP53* loss/mutation, and inactivation of tumor suppressor genes and microRNAs by CpG island methylation [36]. Intestinal metaplasia has been characterized by *TERT* activation, loss of retinoic acid receptor β (*RARB*) expression, *TP53* overexpression, and an abnormal presence of CD44 transcripts. “Gastric adenomas” have been found to exhibit reduced levels of *CDKN1B* expression, overexpression of *CCNE1*, and mutations in the *APC* gene. Furthermore, in cases of advanced stage GC, reduced transforming growth factor β (TGF-β) receptor type I (TβRI) expression and complete loss of function of p27, mutations in tyrosine kinase receptors genes (*EGFR, ERBB2, ERBB3*), amplification of *VEGFA, PIK3CA*, and *KRAS/NRAS*, as well as mutations in chromatin remodeling genes (*ARID1A, MLL3*, and *MLL*) have been observed [4, 6, 36–38].

Diffuse-type adenocarcinoma is not associated with *H. pylori* infection and is hypothesized to arise in normal gastric mucosa that contains a greater number of poorly differentiated cells than intestinal-type cells, yet it does not involve a specific carcinogenic sequence. The main molecular changes observed in diffuse-type GCs include microsatellite instability (MSI), loss of E-cadherin function by deletions or mutations in *CDH1*, amplification of *MDM2* and *MET* and *FGFR2F* and *RHOA* mutations [36, 39, 40]. The main genomic and epigenetic alterations found in intestinal-type and diffuse-type adenocarcinomas are summarized in Figure 1. Currently, it remains unclear whether the molecular alterations associated with the Correa carcinogenic model are sequential, or whether some histological changes directly precede GC development [41]. However, an increasing number of genetic abnormalities have been identiﬁed over the past couple decades, thereby providing additional insight into the molecular alterations that lead to the development of GC subtypes.

## A NEW MOLECULAR CLASSIFICATION OF GASTRIC ADENOCARCINOMA

**Figure 2:**
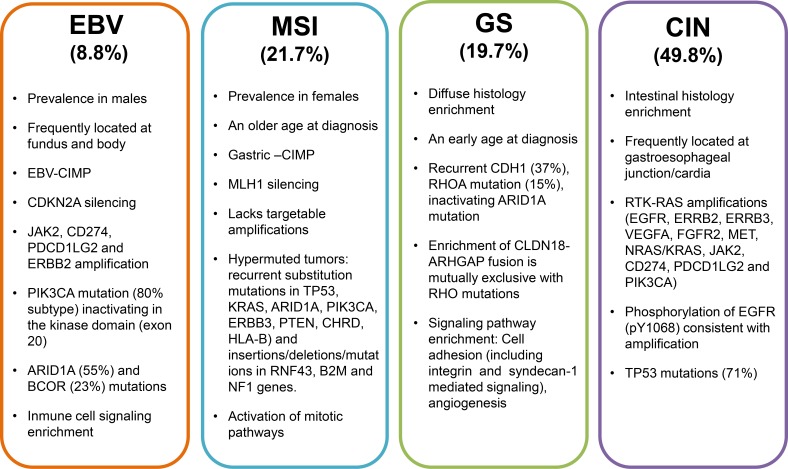
Major features and salient genomic alterations that have been associated with each molecular subtype of GC proposed by the TCGA CIMP: CpG island methylator phenotype; EBV: Epstein-Barr virus; MSI: Microsatellite instability; GS: Genomically stable; CIN: Chromosomal instability.

Researchers of the Cancer Genome Atlas Research (TCGA) network recently examined 295 stomach tumors and identified subtypes using complex statistical analyses of molecular data obtained from six molecular analysis platforms that included DNA sequencing, RNA sequencing, and protein arrays. As a result, they have described a new molecular characterization that defines four major genomic subtypes of GC [4]. The most important features and genomic alterations associated with each subtype are shown in Figure 2, and these are subsequently discussed in the paragraphs that follow.

### Epstein Barr Virus (EBV)-positive subtype

Approximately 9-10% of gastric adenocarcinomas are positive for EBV, and most of these cases involve male patients and the localization of adenocarcinomas in the fundus or body of the stomach [4, 42]. EBV is the etiologic agent of infectious mononucleosis and is responsible for certain cancers, including nasopharyngeal carcinoma and some types of lymphoma [43–45]. In a recent meta-analysis, EBV-associated GC was found to be associated with an infrequent tendency toward lymph node metastasis and a lower mortality rate in different populations [46]. Approximately 80% of EBV-positive tumors harbor mutations in the *PIK3CA* gene [4], which encodes the PI3Kα protein [47]. *PIK3CA* is the second most commonly mutated gene across many cancer types, and was detected in more than 10% of the cancer cases examined by the TCGA [48]. Mutations in *PIK3CA* have been found in 3-42% of the other GC subtypes and in EBV-positive cases. Of particular interest is whether PI3K signaling pathway inhibitors can distinguish EVB-positive versus EBV-negative GC cases. To date, the EBV-positive subgroup of GC cases have showed the highest prevalence of DNA hypermethylation compared with the panel of cancers studied by the TCGA network. Furthermore, promoter hypermethylation of *CDKN2A* has been detected in all EVB-positive samples, and this gene is considered to be one of the most important tumor suppressor genes involved in GC [49]. Amplifications of *JAK2, CD274, PDCD1LG2*, and *ERBB2* have also been detected in EBV-positive GC cases, as well as deletions in *PTEN, SMAD4, CDKN2A*, and *ARID1A*. The JAK/STAT signaling play an important role in proliferation, differentiation and apoptosis and has been intensely investigates in gastric cancer [50] and increased expression of PD-L1 has been correlated with poor prognosis in the GC cases, potentially contributing to immune suppression and evasion [51, 52]. Correspondingly, PI3K inhibitors, JAK2 inhibitors, and PD-L1/2 antagonists may have therapeutic value for the treatment of EBV-positive GC patients.

### Microsatellite instability (MSI) subtype

MSI is one of the main phenotypes of genomic instability that has been associated with relatively older female GC patients. TCGA researchers have reported that this MSI subgroup represents 21% of the tumors studied, while other groups have reported that MSI cases represent 15-55% of GC cases, depending on the number of loci analyzed [53–55]. MSI cases are generally characterized by accumulation of mutations in *PIK3CA, ERBB3, ERBB2*, and *EGFR*; along with mutations present in ‘hotspot’ sites that have been identified in other cancers. Previous studies have reported that the main cause of MSI in GC involves hypermethylation of the *MLH1* promoter, which is one of the genes involved in the DNA mismatch repair system [4, 55]. Depending on the level of MSI, GC cases can be categorized as high or low (MSI-H and MSI-L, respectively). Conversely, tumors without instability at any microsatellite loci are categorized as microsatellite stable tumors (MSS) [56]. The MSI-H phenotype has been strongly associated with intestinal-type adenocarcinomas, and is associated with a better prognosis than MSI-L or MSS tumors [4, 55, 57, 58]. MSI has also been hypothesized to play a role in the alteration of genes related to cell cycle control (*TGFBR2, EPHB2, E2F4*), DNA damage repair (*MRE1, ATR*), and apoptotic signaling (*BAX*) [56, 59, 60].

### Genomically stable (GS) subtype

The GS subgroup represents approximately 20% of the GC cases examined in the TCGA study [4], and it was enriched with diffuse-type adenocarcinomas. The GS subtype is typically diagnosed in younger patients, it has a rather low frequency of *TP53* mutations, and a low degree of aneuploidy compared with chromosomally unstable GC (described below) [4]. Mutations in the *CDH1* and *RHOA* genes have been detected in 13-37% and in 14-25% of diffuse-type GS adenocarcinoma cases, respectively, thereby highlighting the relevance of altered cell adhesion in the carcinogenesis of this histological type [4, 40, 61]. *CDH1* germline mutations are associated with hereditary diffuse GC, poorly differentiated phenotype and poor clinical outcome [62]. In contrast, mutated *RHOA* interacts with other cellular proteins to promote morphological changes and cell migration, functions that may be important for tumor growth [63–65]. Another genetic alteration related to RHOA activity involves the *ARHGAP26* gene, which encodes a GTPase-activating protein that modulates RHOA activation. Interchromosomal translocation between *CLDN18* and *ARHGAP26* has been described, and it is likely that the chimeric product may affect regulation of RHOA. Furthermore, this genomic fusion may also disrupt wild-type *CLDN18*, a tight junction protein that is widely expressed in stomach epithelium and affects cellular adhesion. Interestingly, *CLDN18-ARHGAP26* fusion is mutually exclusive with *RHOA* mutations [4]. Therefore, alterations in the cell adhesion pathways in this GC subtype may facilitate the identification of novel therapeutic targets, particularly those involved in the RHOA pathway.

### Chromosomally unstable (CIN) subtype

Fifty percent of GC tumors are classified as CIN, and this phenotype is mostly associated with the GEJ/cardia. Moreover, 71% of CIN GC tumors have mutations in *TP53*, followed by mutations in *ARID1A, KRAS, PIK3CA, RNF43, ERBB2*, and *APC* genes. Elevated expression of p53 is consistent with the frequency of *TP53* mutations and aneuploidy that are observed for CIN GC tumors. These findings are also partly consistent with the observation that the highest frequency of loss of heterozygosity occurs at the *APC* (36%) and *TP53* (33%) loci [4]. *TP53* alterations have previously been associated with gastric precancerous lesions; thereby suggesting that loss of p53 function may represent an early event in gastric carcinogenesis [66]. Phosphorylation of epidermal growth factor receptor (EGFR) (pY1068) is also significantly elevated in the CIN subtype, consistent with the detection of *EGFR* amplification in this subtype.

Another important feature of the CIN subtype is the frequent genomic amplification of genes that encode receptors of tyrosine kinases (RTKs), which leads to the promotion of aberrant cell growth. However, the gene that encodes the ligand *VEGFA*, is also frequently amplified in this GC subtype, as demonstrated in studies of ramucirumab, a VEGFR2-targeting antibody. In addition, amplifications of cell cycle mediators (*CCNE1, CCND1*, and *CDK6*) have been observed in the CIN subtype. Many of these genomic amplifications are amenable to blockade by therapeutics that are currently available or are in development, particularly inhibitors of cyclin-dependent kinases.

In the past, GC was considered a single disease. However, it is currently segregated into at least four subtypes according to the spectra of genetic alterations that have been identified. Moreover, these alterations have been associated with relevant clinical features such as etiology, gender, age of diagnosis, and anatomical localization. Taken together, these findings highlight the importance of elucidating the different carcinogenic processes that lead to each subtype, as well as the relevant genes and pathways that may be susceptible to therapeutic targeting.

## ONCOGENIC INTRACELLULAR PATHWAYS IN GC AS POTENTIAL THERAPEUTIC TARGETS

By identifying the molecular characteristics of GC, a classification of GC subtypes has been proposed, intracellular pathways that contribute to carcinogenesis have been elucidated, and driver genes have been recognized as potential therapeutic targets. Furthermore, accumulating evidence regarding the molecular abnormalities of GC provide valuable insight into the targets that may be relevant for distinct patient populations, and these remain to be evaluated in clinical trials. In Figure 2, the major features and salient genomic alterations associated with each molecular subtype of GC proposed by the TCGA are summarized. In Table 3, selected clinical trials of novel therapeutic targets for the treatment of advanced stage GC are presented.

**Table 2:** Signaling pathways and genetic alterations that may represent potential therapeutic targets for GC

Name	Mutation, Amplification and Overexpression Profiling	Correlation
**ErbB Pathway**		
**EGFR (ERRB1)**	Overexpressed in 9% [29, [30] and 27.4% [29] of cases Mutated in 5% of cases [69] Amplification in 2.3% [29], 5.4% [69], and 8.0% [67] of cases	Poor prognosis [29, [30], [79, [82]
**ERBB2 (HER2)**	Overexpressed in 9% [28], 9.3% [255], 10.5% [27], 10% [31], 13.6% [30, 256, 257], 23% [256] and 29.5% [257] of cases [69]Mutated in 5% [69] Amplification 8.2% [69, [255], 10.5% [69] and 7.2% [67] of cases Mutated in 10.5% [69] of cases	Poor prognosis [27, [257]
**ERBB3 (HER3)**		
**ERRB4 (HER4)**	Mutated 9.4% [69] of cases	
**VEGF Pathway**		
**VEGFA**	Overexpressed in 81.5% [96], 54.2% [97, [98], and 90.1% [98] of cases Amplification in 7.0% [4] of cases	Early event [96], tumor size, stage, and lymph node metastases [97]
**VEGFC**	Overexpressed in 55.4% [100], 74.9% [98], and 88.0% [99] of cases	Lymphatic invasion [99]
**VEGFD**	Overexpressed in 63.0% [99] of cases	Lymphatic invasion [99]
**PI3K/AKT/mTOR Pathway**		
**PI3KCA**	Overexpressed in 35% [108], 80% [110], and 38,2% [109] of cases	Lymph node and distant metastases [108]
**p-AKT**	Overexpressed in 82% [110], 42% [109], 40% [112], and 68% [111] of cases	Chemoresistance [133], lymph node metastases [109, [111]
**p-mTOR**	Overexpressed in 60% of intestinal-type cases, in 64% of diffuse-type cases [124], and in 71.1% [125] and 79.6% [109] of GC cases	Early gastric cancer [124]
**p-P70S6K**	Overexpressed in 59.5% [258] and 45.5% [109] of cases	
**HGF/MET Pathway**		
**HGF**	Overexpressed in 87.5% [143] and 73.0% [144] of cases	Associated with intestinal-type GC [143]
**MET**	Overexpressed in 42.0% [145], 46.0% [146], 82.0% [147], 26.0% [148], 43.0% [150], 74.0% [151], 71.1% [149], and 73.7% [152] of cases Amplification in 1.7% [153] and 4% [67] of cases	Poor prognosis [149, 151, 152]
**Hedgehog Pathway**		
**PTCH**	Overexpressed in 16.3% [68] and 64.0% [259] of cases	Poor prognosis [204]
**SHH**	Overexpressed in 71.7% [205] and 65.0% [259] of cases	Poor prognosis [196, 204, 205], early event [196]
**SMO**	Overexpressed in 12.0% [68] of cases	
**IHH**	Overexpressed in 20.0% [68] of cases	
**GLI1**	Overexpressed in 69.0% [259] of cases	

**Table 3:** Selected clinical trials that used novel therapeutic targets for the treatment of advanced stage GC

Clinical trial	Agent / Phase	No. of Patients (n)	Treatment	Clinical efficiency*				Condition
**EGFR/ERBB2 pathway**				**ORR (%)**	**mPFS**	**mOS**	***P*-value**	
**ToGA trial** **Bang et al. (2010) [27]**	Trastuzumab, anti-ERBB2 / Phase III	594	X(FU)P	35	5.5	11.1	0.0046	ERBB2-positive advanced stage GC or GEJ cancer
			X(FU)P-T	47	6.7	13.8		
**EXPAND trial** **Lordick et al. (2013) [79]**	Cetuximab, anti-EGFR / Phase III	904	XP	30	4.4	24	0.95	Advanced stage unresectable (M0) or metastatic (M1) GC or GEJ cancer
			XP-Cet	30	5.6	21		
**REAL3 trial** **Wadell et al. (2013) [82]**	Panitumumab, anti-EGFR / Phase III	553	EOX	42	7.4	11.3	0.013	Untreated, metastatic or locally advanced stage GEJ cancer
			EOX-Pan	46	6.0	8.8		
**TyTAN-Asian trial** **Satoh et al (2014) [93]**	Lapatinib, tyrosine kinase inhibitor of EGFR and ERBB2 / Phase III	261	Paclitaxel	9	4.4	11	NS	ERBB2-amplified advanced stage GC
			Paclitaxel-Lap	27	5.4	8.9		
**TRIO-013/LOGIC trial** **Hecht et al. (2013) [94]**	Lapatinib, tyrosine kinase inhibitor of EGFR and ERBB2 / Phase III	545	XELOX	40	5.4	10.5	0.35	ERBB2-positive advanced or metastatic cancer and GEJ cancer
			XELOX-Lap	53	6.0	12.2		
**HERBIS-1 trial** **Kurokawa et al. (2014) [91]**	Trastuzumab, anti-ERBB2 / Phase II	56	S-1P-T	68	7.8	16	-	ERBB2-positive advanced stage GC
**VEGF pathway**								
**AVAGAST trial** **Ohtsu et al. (2011) [101]**	Bevacizumab, anti-VEGF / Phase III	774	X(FU)P	37.4	5.3	10.1	0.1002	Untreated, unresectable locally advanced or metastatic GC and GEJ
			X(FU)P-B	46	6.7	12.1		
**REGARD trial** **Fuchs et al. (2014) [103]**	Ramucirumab, anti- VEGFR2 / Phase III	355	X(FU)P	24	1.3	3.8	0.047	Previously treated advanced stage GC and GEJ cancer
			X(FU)P Ram	49	2.1	5.2		
**RAINBOW trial** **Wilke et al. (2014) [104]**	Ramucizumab, anti- VEGFR2 / Phase III	665	Paclitaxel-placebo	16	2.9	7.4	0.017	Previously treated advanced stage GC and GEJ cancer
			Paclitaxel-Ram	28	4.4	9.6		
**Li J et al. (2013) [105]**	Apatinib, anti-VEGFR2 / Phase II	114	Placebo	0	1.4	2.5	0.01	Chemotherapy-refractory advanced stage metastatic gastric cancer
			Apatinib	10	3.7	4.8		
**PI3K/AKT/mTOR Pathway**								
**GRANITE -1 trial** **Ohtsu et al. (2013) [127]**	Everolimus, mTORC1 inhibitor / Phase III	656	Placebo	2	1.4	4.3	NS	Advanced stage GC
			Eve	5	1.7	5.4		
**HGF/MET Pathway**								
**NCT00719550-Completed Iveson et al. (2014) [155]**	Rilotumumab, Anti-HGF monoclonal antibody / Phase 1b-II	121	Placebo plus ECX		4.2			Unresectable advanced stage or metastatic GC or GEJ cancer
			Ril (15mg/kg)		5.1			
			Ril (7.5 mg/kg)		6.8			
			ECX-Ril		5.7			

ORR: overall response rate; mPFS: mean progression-free survival; mOS: mean overall survival; X(FU)P: Capecitabine or 5-FU and cisplatin; T: Trastuzumab; XP: Capecitabine, cisplatin; Cet: Cetuximab; EOX: Epirubicin, oxaliplatin; Pan: Panitumumab; Lap: Lapatinib; XELOX: Capecitabine, oxaliplatin; S-1P-T: S-1, cisplatin, transtuzumab; B: Bevacizumab; Ram: Ramucirumab; Eve: everolimus; FLP: 5-FU, leucovorin, cisplatin; Ril: Rilotumumab; ECX: epirubicin, cisplatin, capecitabine; Onar: Onartuzumab; Tiv: tivantinib.

* The clinical efficacy was calculated from published data

## ERBB SIGNALING PATHWAYS

There are four ErbB receptors (1-4) and these are members of the RTK superfamily. These receptors localize to the cell surface and have a predominantly regulatory role in nearly every aspect of cell biology. Accordingly, deregulation of these receptors contributes to the development of various cancers, including GC.

## EGFR (ERBB1)

Activation of EGFR (also referred, as ERBB1) has been reported in 9-30% of GC cases [29, 30]. This correlates with 2-8% of GC cases, which are characterized by *EGFR* amplification [67–69] or mutation (5%) [69]. Interestingly, activation of EGFR mainly due to amplification has been observed in 10% of CIN GC cases, while activation of EGFR due to mutations has been observed in 5% of MSI molecular subtypes [4]. In addition, overexpression of EGFR has been associated with advanced stages of GC and an unfavorable prognosis [29, 30]. Binding of different ligands to EGFR, including epidermal growth factor (EGF) and TGF-α, initiates signal transduction cascades that can lead to activation of the mitogen-activated kinase (MAPK) signaling pathway (KRAS/NRAS/RAF/MEK/ERK) and/or the phosphoinositide 3-kinase (PI3K) signaling pathway (PI3K/PTEN/AKT/mTOR). Consequently, EGFR plays a critical role in cell proliferation, differentiation, migration, and survival [69, 70]. Interestingly, RTK-RAS has been reported to be a dominant oncogenic pathway in approximately 40% of GC cases, and genes related with this signaling pathway are mutually to one another in GC [67]. The latter include *EGFR*, *ERBB2*, *KRAS*, *FGFR2*, and *MET,* and these genes are frequently amplified in CIN molecular subtype cases of GC [4].

Different strategies have been developed for the targeting of EGFR (Table 3), including small molecule tyrosine kinase inhibitors and monoclonal antibodies. Regarding the former, antitumor activity was observed in patients with distal esophageal and GEJ adenocarcinomas that were treated with erlotinib, but the same activity was not observed in patients with distal gastric tumors [71]. In other studies, treatment with gefitinib demonstrated biologic activity against EGFR, yet a comparable response and OS rate was observed for the treatment with gefitinib or erlotinib [72, 73].

Of the studies performed for EGFR-targeting monoclonal antibodies, three recombinant antibodies have been evaluated in clinical trials. Cetuximab is a recombinant human/mouse chimeric monoclonal antibody against EGFR and it represents the best-characterized anti-EGFR therapy for GC to date. In a phase II study that evaluated the use of cetuximab as a single agent for the treatment of patients with metastatic GC, minimal clinical activity was observed, and this was characterized by a response rate of 3%, a stable disease rate of 6%, and a median survival period of 3.1 months [74]. In contrast, when cetuximab was used as a first-line treatment in combination with various chemotherapy regimens in several phase II studies, a tendency for improvement in treatment response was observed in a subset of GC patients, with the overall response rates varying from 40-60% [75–78]. However, in the EXPAND trial [79], an open-label, randomized phase III trial that assessed the combination of cetuximab with XP chemotherapy for patients with advanced stage gastric or GEJ cancer, the median PFS period was 4.4 months (*n* = 455, 95% CI: 4.2-5.5) compared with 5.6 months (*n* = 449, 95% CI: 5.1-5.7) for the patients who received XC chemotherapy alone (*p* = 0.32). Thus, the addition of cetuximab to XP chemotherapy did not provide additional benefit to chemotherapy alone for the first-line treatment of advanced stage GC patients.

Matuzumab and panitumumab are two other anti-EGFR monoclonal antibodies that have been evaluated in clinical trials. Matuzumab first emerged as a promising drug based on the results of a phase I study for the treatment of patients with solid tumors [80]. However, the efficacy of matuzumab for GC has not been demonstrated. In a recent phase I clinical trial conducted by Trarbach et al., the use of matuzumab in combination with 5-FU, leucovorin, and cisplatin for the treatment of advanced stage GC patients was investigated. Matuzumab demonstrated an acceptable safety profile with modest anti-tumor activity, yet the confirmed overall response rate was low (26.7%) [81]. Similar findings were reported for panitumumab in the REAL3 trial, a randomized multicenter phase III study where the clinical efficacy of an epirubicin, oxaliplatin, and capecitabine (EOX) regimen with or without panitumumab was evaluated for the treatment of previously untreated advanced esophagogastric cancer. The addition of panitumumab significantly reduced the median OS period from 11.3 months for the patients that received EOX (95% CI: 9.6-13.0) to 8.8 months (95% CI: 7.7-9.8) for the patients that received panitumumab plus chemotherapy. Furthermore, EOX plus panitumumab was associated with increased grade 3 and 4 adverse effects, requiring dose reductions. The anti-EGFR phase III trials EXPAND and REAL-3, for cetuximab and panitumumab respectively, failed to meet their primary endpoints, casting a large shadow over future prospects for other anti-EGFR drugs [83]. However, in both trials EGFR-overexpressing patients were not preselected as an inclusion criterion in the trial, and later subgroup analyses may be effective in the subgroup of patients that highly express EGFR [79]. Another important aspect to highlight in the failure of anti-EGFR targeted therapy are the intra-tumor heterogeneity, acquired resistance to anti-EGFR inhibitors and the non-existence of an established biomarker to predict response to anti-EGFR treatments [84, 85]. Acquired resistance to anti-EGFR inhibitors may result from activation of partner of HER family (HER3 and/or HER2), which share overlapping signaling pathways [86]. In colon cancer studies, *RAS* mutations (*KRAS* and *NRAS*) have been investigated as predictive markers of anti-EGFR treatments. In contrast, RAS mutation is only observed in 5% of GC patients [87], and there has been no definite evidence of its mutation playing a role as a predictive factor for anti-EGFR antibody therapy.

Taken together, these finding suggest that an EGFR-targeting agent alone is not effective in all patients with GC and there may be a preferred chemotherapy partner for the EGFR antibody [86]. Furthermore, additional clinical trials should assess the activity of EGFR-targeting inhibitors according to the different molecular subgroups of advanced stage GC, including subtyping for EGFR amplification.

## ERBB2 (HER2)

Overexpression of ERBB2 (which is also commonly referred to as HER2) has been detected in 10-30% of GC cases, with amplification of *ERBB2* detected in 2-27% of GC cases [28–30] and *ERBB2* mutations detected in 5% of GC cases [69]. Overexpression of ERBB2 has also been associated with *ERBB2* amplification in 24% of CIN GC cases and in 12% of EBV cases, while *ERBB2* mutations have been detected in 7% of MSI molecular subtypes [4]. Moreover, overexpression of ERBB2 has been associated with poor prognosis and more aggressiveness disease [28].

To date, targeting of ERBB2-overexpressing tumors with trastuzumab has been the most successful example of a targeted agent used for the treatment of GC [88]. Trastuzumab is a recombinant humanized monoclonal antibody that is designed to target and block ERBB2 by inhibiting dimerization, by inducing antibody-dependent cellular cytotoxicity, and by increasing receptor endocytosis [89, 90]. Trastuzumab was the first drug successfully used to treat ERBB2-amplified, advanced stage GC as demonstrated in the Trastuzumab for Gastric Cancer (ToGA) trial [27]. In this phase III multicenter, randomized controlled study, the efficacy of two first-line chemotherapy regimens consisting of trastuzumab combined with standard chemotherapy (XC or 5-FU plus cisplatin) versus the use of chemotherapy alone was examined for patients with inoperable, locally advanced, recurrent or metastatic ERBB2-positive gastric cancer. Patients in the trastuzumab group had a longer OS period than those who received chemotherapy alone (13.8 months *vs*. 11.1 months, respectively). Treatment with trastuzumab also improved the median PFS period (6.7 months *vs*. 5.5 months, respectively) and the radiological response rate (47% *vs*. 35%, respectively). Similar results were obtained in a Phase II study that evaluated the use of trastuzumab in combination with cisplatin in patients with untreated ERBB2-positive advanced gastric or gastroesophageal junction cancer [31]. In a recent phase III trial that combined the use of trastuzumab with S-1 plus cisplatin, a response rate of 68% (95% CI: 84-94%) and median OS and PFS periods of 16.0 months and 7.8 months, respectively [91]. Taken together, these results suggest that trastuzumab represents a new therapeutic option for patients with ERBB2-positive advanced stage GC. To further investigate the role of other ErbB receptors, a phase I trial is recruiting patients to evaluate a treatment regimen including trastuzumab and LJM716, a ERBB3 protein inhibitor (NCT01602406) [92]. ERBB3 appears to be activated by mutations in 14% of MSI GC cases, while amplifications of ERBB3 have been detected in 8% of CIN molecular subtypes of GC [4, 69].

Another drug that was recently described with positive results against ERBB2-positive GC is lapatinib, an intracellular tyrosine kinase inhibitor of EGFR and ERBB2 that acts by blocking autophosphorylation and downstream signaling. The combination of lapatinib with paclitaxel showed anti-tumor activity as a second-line treatment for patients with ERBB2 FISH-positive IHC 3+ advanced stage GC, yet it did not significantly improve the OS of the intent-to-treat population [93]. In the TRIO-013/LOGIC trial, a first-line treatment for ERBB2 overexpression due to gene amplification, advanced stage GC patients was evaluated with lapatinib combined with capecitabine and oxaliplatin (e.g., the XELOX regimen) in 545 patients. PFS, but not OS, was found to be improved [94]. The non-survival benefit shown by Lapatinib combination therapies has been correlated with serious adverse effects such as diarrhea and skin toxicity [93, 94]. Currently, a phase I clinical trial to evaluate the combination the lapatinib with trastuzumab in locally advanced or metastatic GC is in the process of being reported (NCT01705340) [92].

Currently, ERBB2-positive patients with advanced stage GC receive a standard therapy of capecitabine or 5-FU with cisplatin and trastuzumab. To date, the role of lapatinib, alone or in combination with trastuzumab, appears to be promissory, although additional clinical trials are necessary. An accurate molecular characterization of ERBB2-positive tumors is also necessary in order to define which patient groups are likely to benefit from a targeted therapy, thereby improving the cost-effectiveness and efficacy of GC treatment.

## THE VEGF PATHWAY

Angiogenesis is defined as the formation of new blood vessels from pre-existing vasculature and is considered a hallmark of cancer. Angiogenesis also involves the proliferation and migration of endothelial cells into nutrient-deprived tissues, especially into regions adjacent to a tumor where the formation of patent blood vessels is initiated [95].

VEGFA is a member of the PDGF/VEGF growth factor family and it encodes a protein that is often found as a disulfide-linked homodimer. VEGFA acts specifically on endothelial cells and mediates various effects, including increased vascular permeability, angiogenesis, vasculogenesis, and endothelial cell growth, thereby promoting cell migration and inhibiting apoptosis. VEGFA overexpression has been reported in 54-90% of GC cases, and has been described as an early marker in the development of GC [96–98]. Furthermore, expression of VEGFA has been found to correlate with lymph node metastasis and poor prognosis. The growth factors, VEGFC and VEGFD, are also overexpressed in 50-80% of GC cases, and high levels of expression correlate with lymphatic invasion [99, 100]. Interestingly, recurrent amplification of VEGFA has recently been reported to be a trait of the CIN subtype of GC, and this subgroup of cases may be candidates for VEGF-targeting therapies [4].

Anti-angiogenesis therapies have been well-studied for cases of advanced stage GC (Table 3). For example, in the multinational, placebo-controlled phase III trial, Avastin in Gastric Cancer (AVAGAST), the efficacy of adding bevacizumab to a XP protocol for the first-line treatment of advanced stage GC was examined. Unfortunately, AVAGAST did not accomplish its primary endpoint of extending the OS of patients with GC [101]. However, subgroup analyses demonstrated that significantly longer OS periods were achieved for patients from non-Asian regions [17, 102]. Ramucirumab, a monoclonal VEGFR2 antagonist, is the first FDA-approved biological therapy for the treatment of advanced stage GC that is unresponsive to fluoropyrimidine or platinum-containing chemotherapy. In a phase III trial where remucirumab was administered as a single drug for patients with advanced stage GC, the OS period for the patients that received ramucirumab versus a placebo was 5.2 months and 3.8 months, respectively (*P* = 0.047) [103]. Furthermore, in the RAINBOW trial, paclitaxel plus ramucirumab versus paclitaxel plus placebo were compared for the treatment of advanced, pretreated cases of GC. The results of this trial confirmed the survival advantage of ramucirumab plus paclitaxel for the treatment of GC in non-Asian population [104]. The absence of survival benefit in the RAINBOW and AVAGAST in the Asian patient subset could be explained by the overall survival in patients from Asia being extremely longer than non-Asian patients, independent of treatment. It was difficult to obtain the survival benefit in Asian patients with good performance status and subsequent therapies compared with the rest of the world. Moreover, the differences between Asian and Western patients could have affected the results, for example the proportion of different subtypes of molecular groups, the proportion of GC related to EBV, the expression of polymorphism of interleukin or the expression of distinct tumor immunity signatures related to T-cell function.

Others agents that have recently been evaluated in phase II trials include apatinib, a VEGFR2 inhibitor, and the multi-targeted tyrosine kinase receptor inhibitor, sorafenib. For cases involving chemotherapy-refractory advanced stage metastatic GC, treatment with apatinib improved the OS and PFS of this cohort [105]. In a previous study, sorafenib treatment combined with chemotherapy resulted in a median OS period of 13.6 months and a median PFS period of 5.8 months in patients with metastatic or advanced stage GC and GEJ cancer [106]. Based on these results, additional studies of these inhibitors in combination with chemotherapy are required.

## THE PI3K / AKT / MTOR SIGNALING PATHWAY

**Figure 3:**
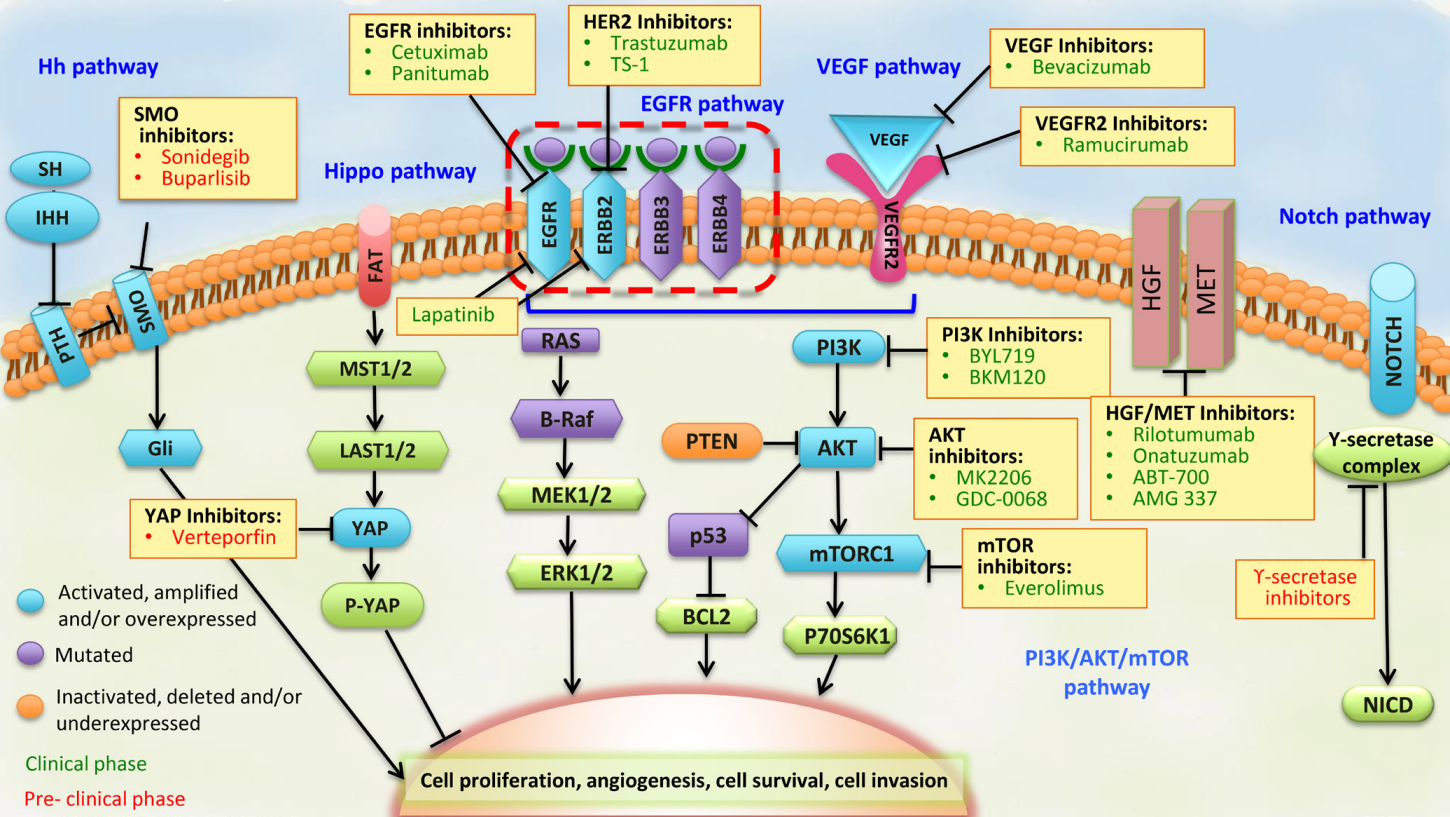
Pathways that represent potential targets for the treatment of advanced stage GC The components of each signaling pathway are colored according to their dominant alteration type (see key at lower left). Targeted agents (listed in yellow boxes) include those in clinical use (colored in green) and those in preclinical or early phase development (colored in red) for the treatment of advanced stage GC. BCL2, associated agonist of cell death; EGFR, epidermal growth factor receptor; NICD, NOTCH intracellular domain; PTCH, Patched; SMO, Smoothened; SSH, slingshot.

The PI3K family of intracellular kinases mediates the regulation of cell survival, proliferation, differentiation, migration, and metabolism [107]. In particular, subunit p110α of PI3K is downstream of activated RTKs, such as EGFR and ERBB2, is an activator of AKT, and is also a downstream effector of the mammalian target of the rapamycin (mTOR) pathway (Figure 3). Activation of the PI3K/AKT/mTOR pathway can be triggered by the activation of RTKs, *PI3KCA*-activating mutations and amplifications, loss of *PTEN* function due to deletions or mutations, and overexpression or activating mutations of *AKT1*.

The PI3K/AKT/mTOR pathway is frequently activated in GC, with overexpression of *PI3KCA* described in 35-80% of GC cases [108–110], and phosphorylation of AKT described in 40-82% of GC cases [109–112]. Expression of PI3KCA and phosphorylated AKT has also been associated with lymph node metastasis [108, 109, 111]. Furthermore, alterations in *PIK3CA* have been detected in 80% and 42% of the EBV and MSI molecular subtypes of GC, respectively [4].

To date, two classes of PI3K inhibitors have been evaluated for the treatment of GC: pan-PI3K inhibitors, which target all PI3K family members (BKM120, PX-886, and XL147), and isoform-specific PI3K inhibitors, which specifically target the p110 catalytic subunit of PI3K (BYL719, GDC0032, and INK1117) [113]. In particular, the pan-PI3K inhibitor, BKM120, has been evaluated in solid tumors, including GC tumors, in combination with LDE225, a hedgehog pathway inhibitor (NCT0157666) [92]. Currently, the isoform-speciﬁc p110α inhibitor, BYL719, and the heat shock protein 90 inhibitor, AUY922, are being evaluated in a phase I trial with advanced stage GC patients that carry either a molecular alteration of PIK3CA, or *ERBB2* ampliﬁcation (NCT01613950) [92]. Due to the structural similarity of the catalytic domains of p110 and mTOR, dual PI3K/mTOR inhibitors (BEZ235, XL765, GDC-0980, GDC0084, SF1126, and PF-46915) have also been developed [114]. These dual PI3K/mTOR inhibitors have been shown to enhance 5-FU cytotoxicity both *in vitro* and *in vivo* [115], especially in PI3KCA mutant gastric tumor cells which are thought to be secondary to cellular heterogeneity in regard to sensitivity to PI3K and mTOR inhibition [116].

Two types of AKT inhibitors been evaluated in clinical trials: allosteric inhibitors (MK-2206) and catalytic site inhibitors (AZD5363, GSK690693, and GDC0068). The allosteric inhibitors of AKT, have been effective against breast cancer cell lines carrying *PI3KCA* mutations and *ERBB2* ampliﬁcations [117]. Currently, MK2206 is being studied in early phase trials in mutation-selected and unselected patients with advanced stage gastric or GEJ cancers or other solid tumors (NCT01260701) [92]. Of the catalytic site inhibitors, AZD5363 has shown activity against GC cell lines *in vitro* [118, 119], and, as a monotherapy, has mediated partial responses in two patients that harbored tumor mutations in either *AKT1* or *PI3KCA* [120]. Similarly, GDC0068 has exhibited anti-tumor activity in human cancer cell lines and xenograft models [121], and is being investigated in a multicenter phase II trial of gastric and GEJ cancer patients with locally advanced or metastatic disease (NCT01896531) [92].

The mTOR complex-1 (mTORC1) inhibitor, everolimus, has been used to successfully treat several cancer types [122, 123]. For GC, everolimus has been tested in phase II and III clinical trials of advanced stage and metastatic GC patients, since activation of mTOR has been reported to occur in 60-80% of GC cases [109, 124, 125]. In a recent phase II trial, everolimus demonstrated a response rate of 3.7% (2/44) and a disease control rate (DCR) of 38.9% (17/44) [126]. However, in a phase III GRANITE-1 study which evaluated the efﬁcacy of everolimus compared to the best supportive care available for molecularly unselected patients with advanced stage GC that progressed after previous chemotherapy, improved survival was not significantly observed (5.39 months with everolimus versus 4.34 months with placebo, HR, 0.90; 95% CI: 0.75 - 1.08; *P* = 0.124) [127]. It is possible that since everolimus only suppresses mTORC1, feedback activation of MAPK may limit the anti-tumor potency of everolimus [128]. Correspondingly, there are a few inhibitors that target both mTORC1 and mTORC2 that have exhibited improved potency (OSI-027, BEZ235, XL765, AZ8055, Ink128) and are currently being evaluated in phase I/II trials for patients with other types of solid tumors.

The molecular mechanisms involved in sensitivity to PI3K inhibitors are yet to be clarified in order to translate preclinical activity into clinical benefit, and to date the development of PI3K inhibitors in advanced GC is still in the preclinical stage [129]. Junk et al. [130] observed that 78% of patients with colorectal cancer with the *PIK3CA* mutation also had simultaneous *KRAS* mutation and did not respond to PI3K/AKT/mTOR axis therapy. By contrast, patients with ovarian cancer and simultaneously occurring *KRAS* or *BRAF* mutations achieved a partial response with these inhibitors, thereby indicating that the RAS/RAF/MEK pathway serves as a driver of resistance to PI3K inhibitors and suggesting that screening for *PI3KCA* (and *RAS* or *RAF*) could be used to predict PI3K/AKT/mTOR response clinically. In gastric cancer, the PIK3CA mutation is an important biomarker for predicting the treatment response of everolimus and AKT inhibitors [118, 131]. In contrast, the predictive ability of *KRAS* and *BRAF* mutations has not been extensively studied, but a few reports have demonstrated that the frequency of these concurrent aberrations is very low [87, 118]. Moreover, several studies have implicated the PI3K/AKT/mTOR pathway in mediating resistance by GCs to chemotherapy and anti-ERBB2 treatment [132–134]. It is hypothesized that AKT affects the BCL2 protein and the NF-κB pathway, although PI3K may also induce upregulation of the chemo-resistance proteins, MDR1/Pgp, BCL2, and XIAP, while downregulating the expression of BAX and caspase 3. In tumor tissues from GC patients that were examined *in vitro*, AKT activation and *PTEN* loss were associated with increased resistance to multiple chemotherapeutic agents (5-FU, doxorubicin, mitomycin C, and cisplatin) [135]. Similarly, a combination of PI3K and AKT inhibitors with chemotherapy agents has successfully attenuated chemotherapeutic resistance in a synergistic manner in GC cell lines [134, 136] and other cancer models [121], especially those characterized by *PTEN* loss [118]. A similar association was observed following activation of the PI3K/AKT pathway and resistance to anti-ERBB2 agents in other cancers [118, 119]. Based on the observation that *PTEN* loss was detected in a majority of ERBB2-positive GC cases [137], it is possible that *PTEN* loss explains the observed clinical resistance of ERBB2-positive GC patients to current anti-ERBB2 therapies.

## THE HGF/MET SIGNALING PATHWAY

This pathway is characterized by the joint action of two proteins: hepatocyte growth factor (HGF) (also referred to as scatter factor) and its only known receptor, MET. These proteins regulate multiple cellular processes that stimulate cell proliferation, migration, invasion, angiogenesis, apoptosis, and metastasis, thereby leading to the activation of MAPK, PI3K-AKT, v-src sarcoma viral oncogene homolog, and signal transducer and activator of transcription (STAT) signaling pathways [138–140]. Recent evidence has also highlighted the additional roles for the MET in cancer via crosstalk with other receptors and cell surface proteins, such as *TGFB1* and *EGFR* [141], and these interactions contribute to oncogenesis and drug resistance [142].

Overexpression of HGF and MET [143, 144] have been reported in 73-88% and 26-82% [145–152] of advanced stage GC cases, respectively. Furthermore, overexpression of MET has been associated with poor prognosis in advanced stage GC cases [149, 151, 152]. Inappropriate MET signaling in cells can be triggered by several mechanisms. The first is related to a *MET* rearrangement generated by chromosomal translocation and fusion of a TPR (translocated promoter region) locus on chromosome 1 to the 5′region of sequences derived from the *MET* locus on chromosome 7. This genomic rearrangement results in activation of MET via dimerization of its kinase domain and allows MET to escape the normal mechanisms of down-regulation. The *TPR-MET* chromosomal translocation has been found in precursor lesions of GC and in adjacent normal mucosa [139]. Other genetic mechanisms that could be related to GC lesions include gene amplifications, gene mutations, and transcriptional up-regulation of *MET* and/or *HGF* genes. Correspondingly, *MET* gene amplification with consequent protein overexpression and constitutive kinase activation has been reported in 2-4% [67, 153] of advanced stage GC cases, amplifications of *MET* have been detected in 8% of CIN GC lesions, and *MET* mutations are present in approximately 3% of MSI subtype GCs [4].

MET is a popular target and several clinical trials are underway to evaluate monoclonal antibodies, such as rilotumumab (AMG102), onartuzumab (MetMAb), ABT-700 as well as MET-specific and multi-targeted small-molecule tyrosine-kinase inhibitors, for the treatment of GC. Specifically for the treatment of GEJ and GC in the later stages of clinical development, the monoclonal antibodies, tivantinib, AMG 337, foretinib, cabozantinib, and golvatinib, are also being investigated [139].

Rilotumumab is a neutralizing monoclonal antibody that prevents the binding of HGF/SF to the MET receptor and its subsequent signaling [154]. In a phase II study of rilotumumab in combination with epirubicin, cisplatin, and capecitabine (ECX) for the treatment of advanced stage or metastatic gastric and GEJ cancers, an improvement in OS and PFS was observed for the patients that received rilotumumab plus ECX [155]. It is hypothesized that high MET expression detected by immunohistochemistry may predict a clinical benefit from rilotumumab plus ECX for GC patients, and may also be associated with a poor prognosis for ECX-treated patients. These possibilities are being addressed in the terminated RILOMET-1 phase III trial that includes MET-positive GC and GEJ patients (NCT01697072) and the final results are expected to be published [92]. Onartuzumab (MetMAb) is an *Escherichia coli*-derived, humanized monovalent (one-armed), monoclonal antibody against MET [156]. In contrast, the monovalent design of onartuzumab inhibits HGF/SF binding without inducing MET dimerization [157]. When onartuzumab was administered by intravenous infusion as a single agent or in combination with bevacizumab in patients with advanced solid malignancies as part of a phase I study [158], promising results were obtained. Therefore, onartuzumab has been advanced to phase II and III studies in combination with FOLFOX to treat patients with metastatic ERBB2-negative solid tumors, including GC. The results of these studies are expected soon (NCT01590719 and NCT01662869) [92]. Another important monoclonal antibody is ABT-700, which has shown promising anti-tumor activity in patients with *MET* amplification solid tumors [159]. The ABT-700 was well tolerated at the recommended single-agent dose of 15mg/kg without important adverse events. Furthermore, a phase I study using ABT-700 monotherapy showed a 50% response rate in patients with *MET*-amplified advanced gastric and esophageal cancer (NCT01472016) [160]. Interestingly, the *MET* amplification appears to be more common in metastatic recurrent tumors than in primary tumors and this highlights the importance of identifying *MET* amplification as a predictive biomarker of clinical benefit in a treatment-refractory patient population.

To date, only a few MET inhibitors have undergone clinical trials. Tivantinib is a small molecule that represents an orally bioavailable MET inhibitor that blocks the MET receptor in its non-phosphorylated, inactive conformation and interrupts downstream signaling [161]. In a recent phase II trial for tivantinib, modest efficacy was observed in an Asian patient that was previously treated for metastatic GC [162]. Currently, a phase I-II trial is being conducted to evaluate combinations of tivantinib and FOLFOX, pazopanib, and bevacizumab for the treatment of patients with GEJ and gastric cancer (NCT01611857, NCT01468922 and NCT01749384) [92]. The novel AMG 337 MET tyrosine-kinase inhibitor looks promising and points towards a potentially effective new treatment option for a new patient subgroup with MET-amplified gastric, esophageal and GEJ tumors [163]. In a phase I analysis of AMG 337 monotherapy, 14.4 % (13/90) of patients showed *MET* amplification. Among these 13 patients, 62% (8/13), achieved an objective response to AMG 337. The daily maximum tolerated dose was 300 mg and in regard to safety the most common adverse events were nausea, vomiting and fatigue, and the only dose-limiting toxicity was headache [164]. On the basis of these results, a phase II study is currently recruiting patients (NCT02016534) [92]. In addition, foretinib (GSK1363089, XL880), an oral multikinase that primarily targets MET, RON, AXL, and VEGFR, is also being investigated [165]. In preclinical studies, foretinib prevented tumor growth by mediating a direct effect on cell proliferation and by inhibiting cell invasion and angiogenesis that involved the HGF and VEGF receptors [166]. In a phase II trial of metastatic GC patients, administration of foretinib alone did not provide sufficient efficacy in unselected patients with metastatic GC and this absent of response could be due to the evaluation of non-molecularly selected population [167]. Other tyrosine kinase inhibitors that have been examined include cabozantinib (XL184) and golvatinib (E7050). The former is a multikinase inhibitor that targets MET, VEGFR2, AXL, Tie2, KIT, FLT3, and RET [168]. In 2012, the FDA approved cabozantinib for the treatment of patients with metastatic medullary thyroid cancer, and a phase II clinical trial for the treatment of advanced stage malignancies by cabozantinib, including gastric and GEJ cancer, is ongoing (NCT00940225) [92]. Golvatinib (E7050) is an ATP-competitive inhibitor of MET receptors that potently and selectively inhibits the autophosphorylation of MET and VEGF-induced phosphorylation of VEGFR. When golvatinib in combination with gefitinib was used to treat EGFR-mutated lung cancer cell lines, the MET/Gab1/PI3K/AKT pathway was blocked [169]. Golvatinib was subsequently evaluated in a phase I study in patients with advanced stage solid tumors [170]. Currently, studies of golvatinib (E7050) in combination with other targeted agents, including sorafenib, and with lenvatinib (E7080) in patients with advanced stage malignancies, are ongoing. These results, as well as those of ongoing studies, are of great interest regarding this promising pathway in GC.

## THE RHOA SIGNALING PATHWAY

Rho GTPases are important intracellular signaling molecules that regulate cytoskeleton organization, cell cycle, and cell motility, among other processes. In cancer, Rho activity promotes metastasis by disrupting the epithelial layer, by fostering motility, and by inducing degradation of the extracellular matrix [171]. In recent studies, well-described *CDH1* mutations, as well as *RHOA* mutations, have been found to be strongly related to histologic diffuse-type gastric carcinogenesis [40, 61], and this in turn, enriches the GS subgroup of GC [4]. These results are consistent with the hypothesis that diffuse-type GC is a neoplasm whose initial existence relies more on the inherent characteristics of the tissue than on disorganization of the genome, compared with the other subtypes [62]. The diffuse-type subtype is also characterized by the early breaking-off of signet-ring cells through the basement membrane, and this process requires resistance to anoikis (e.g., apoptosis induced by a lack of correct cells to extracellular membrane attachment) and is characterized by an infiltrative phenotype. In three-dimensional cultures, mutated *RHOA* has been associated with an ability to promote escape from anoikis and to facilitate tumor growth, thereby implicating RHOA as an oncogenic driver of diffuse-type GC progression [40, 61]. Rhosin and Y16 represent newly developed Rho-specific inhibitors that potently suppress breast cancer cell proliferation, migration, and invasion *in vitro* [172]. However, due to their distinct mechanisms, they are able to act synergistically to inhibit RHOA-mediated cell stress fiber formation [172–174]. Ripasudil, a selective inhibitor of Rho-associated coiled coil-containing protein kinase (ROCK) was approved in Japan in September 2014 for the treatment of glaucoma and ocular hypertension [173]. Accordingly, it is predicted that current and future drugs that inhibit the RhoA pathway will be evaluated in clinical trials in patients with GC.

## THE JAK/STAT SIGNALING PATHWAY

Janus-associated kinase 2 (*JAK2*) is overexpressed in a subset of EBV-subtype GCs, and the JAK/STAT signaling pathway has been detected in several types of tumors, including GC [50, 175]. Therefore, JAK2 inhibitors may also represent a potential therapeutic treatment for GC. JAK2 is potentially the most influential kinase in this family based on its interactions with growth hormone receptors and cytokine receptors. Following the activation of JAK2 by phosphorylation, STAT phosphorylation is induced and gene expression involved in cell proliferation and apoptosis arrest is stimulated [176]. Inhibition of JAK2 has been applied to myeloproliferative disorders such as polycythemia vera, essential thrombocythemia, and primary myelofibrosis [177]. For multiple myeloma patients, inhibition of JAK2 led to a significant reduction in splenomegaly, an elimination of debilitating disease-related symptoms, and weight gain [178]. JAK2 inhibiting drugs include: INCB018424 (phase III), TG101348 (phase II), and CEP701 (phase I/II) [178, 179]. Other JAK2-inhibiting molecules have been investigated as well. For example, AZD1480 was tested in a phase I study of 38 patients with advanced stage solid tumors, and rapid absorption and elimination of this drug was observed (NCT01219543) [92]. However, the authors could not establish whether dose-limiting toxicities were due to inhibition of JAK1/2 or were due to off-target effects [180]. AG-490 has been tested on SGC7901 and AGS gastric cell lines, and inactivation of JAK2 and induction of apoptosis were observed in the latter [181]. Finally, WP-1066 was assessed in AGS cell lines and in gp^757FF^ mice as models of GC. In both models, cell proliferation was blocked, inflammation was reduced, and apoptosis was induced in gastric tumor cells by inhibiting STAT3 phosphorylation. While clinical studies are needed to confirm these promising results in patients with advanced stage GC, WP1066 may form the basis for the development of future therapeutics against GC [182].

## STEM CELL PATHWAYS PROVIDE NOVEL GASTRIC CANCER THERAPY TARGETS

Gastric cancer stem cells (GCSCs) have been identified in GC cell lines and primary GC tissues [183–186]. Moreover, recent investigations have shown that gastric GCSCs are a crucial target for GC treatments [187–189], with strategies targeting self-renewal pathways leading to the direct elimination of cancer stem cells (CSCs). Stemness-related signaling pathways include Hedgehog (Hh), Wnt, Notch, and Hippo, and these have been widely implicated in the maintenance of CSCs [190, 191]. Considering that conventional chemotherapy only acts on active cells, and not on quiescent CSCs, targeting of characteristic signaling pathways of CSCs may represent a promising approach for GC therapy [191].

## THE HEDGEHOG (HH) SIGNALING PATHWAY

Sonic Hh molecules (Sonic, Indian, and Desert Hedgehog) are produced in gastric epithelial cells and they play a crucial role in the development and regulation of gastric epithelial cell differentiation and regeneration. They also have an important role in the maintenance of adult stem cells [191–194]. The Hh signaling pathway has recently been recognized as one of the most important signaling pathways in the cell, and thus, is a therapeutic target in cancer. In adults, mutations or deregulation of this pathway have been found to play a key role in both cell proliferation and differentiation, thereby leading to tumorigenesis or accelerated tumor growth in a wide variety of tissues. In addition, recent findings suggest that Hh signaling may also promote tumorigenesis in a paracrine manner from the tumor to the surrounding stroma, or in CSCs [195]. Overexpression of Patched (PTCH1), sonic Hh (SHH), Smoothened (SMO), Indian, GLI1, and GLI2 have been found with great frequency in GC cases (Table 2). In particular, the SHH ligand has been found to be deregulated during the progression from inflammation to intestinal-type cancer, indicating that the Hh protein is involved in the early stages of gastric carcinogenesis [196, 197]. *In vitro*, Kim et al. [198] have demonstrated that overexpression of SHH and GLI1 occurs in a *H. pylori* CagA-dependent manner partly through activation of the NF-κB pathway in GC cells. Subsequently, they reported that GLI1 overexpression suppressed Wnt transcriptional activity and nuclear β- β-catenin accumulation, and these pathways contribute GC cell differentiation [199]. Crosstalk between the Hh and Wnt pathways was initially suggested by Yanai et al. [200] who reported the critical roles of these two pathways in the progression of GC. Interestingly, the Hh pathway may mediate crosstalk signaling with the KRAS-MEK-ERK signaling pathway in the regulation of cell proliferation in GC [201]. Emerging evidence further suggests that the SHH pathway is not only involved in the development of GC, but also in the progression, aggressiveness, and metastasis of this disease [202, 203]. Recently, expression of SHH, PTCH1, and GLI2 were found to be independently associated with poor survival [68, 204, 205]. In addition, Yoo et al. elucidated that the SHH pathway mediates cell invasion, metastasis, and lymphangiogenesis via activation of AKT, the epithelial-mesenchymal transition, and the MMP9 pathway in GC [203].

Several proteins in the Hh signaling pathway have been identified as potential drug targets for inhibiting aberrant Hh signaling. The first identified Hh pathway inhibitor, cyclopamine (a SMO inhibitor) has low affinity and poor bioavailability, and thus, more potent derivatives have been synthesized. Of the potential Hh signaling pathway targets, only the SMO antagonists have been tested in humans, including vismodegib, IPI-926, LDE-225, BMS-833923, and PF-04449913. In the initial studies of GC cell lines treated with cyclopamine and Gli transcription factor inhibitor (GANT61), cell survival, proliferation, migration, and colony formation were inhibited [206–208]. *In vivo* studies have subsequently demonstrated that cyclopamine significantly prevents tumor growth and development [208], while preclinical assays that combined chemotherapy with vismodegib (GDC-0449, SMO inhibitor) to treat a subpopulation of CD44+ gastric tumor stem cells were found to reverse resistance to conventional chemotherapy [209].

Vismodegib was approved by the FDA in 2012, particularly as a treatment for advanced stage and metastatic basal cell carcinoma (BCC) and for select patients with medulloblastoma [210, 211]. However, in a phase II clinical trial of patients with metastatic colorectal cancer, treatment with vismodegib exhibited no incremental benefit in combination with FOLFOX [212] or compared with standard FOLFOX or FOLFIRI plus bevacizumab therapies [213]. Currently, the clinical roles of vismodegib, LDE-225, and BMC-833923 in recurrent and metastatic advanced stage gastric adenocarcinomas are been evaluated. The results of two phase I/II clinical trials of SMO inhibitors in combination with conventional cytotoxic therapy, or with a PI3K inhibitor, for the treatment of advanced stage GC (BKM120) are also awaited (NCT00982592 and NCT01576666) [92]. Another interesting aspect is the recent observation that crosstalk and cooperation occurs between the Hh and Wnt, MAPK, and PI3K/AKT signaling pathways [200, 201, 203]. These results are of particular interest because agents that selectively inhibit these pathways are available and can be readily combined with agents such as vismodegib, LDE225, and BMS-833923 [214].

## THE WNT SIGNALING PATHWAY

The Wnt signaling pathway regulates cell proliferation, survival, morphology, migration, self-renewal in stem cells, and specification of cell fate during embryonic development. Wnt signaling also plays an important role throughout adult life by maintaining the homeostasis of tissues via the regulation of somatic stem cells in their niches [215–217]. Currently, Wnt signaling is divided into canonical and non-canonical pathways. In the former, Wnt signals (extracellular ligands, such as wnt-1) stabilize β-catenin, thereby leading to the activation of gene transcription based on interactions between β-catenin and transcription factors [218]. Accordingly, abnormal activation of the Wnt/β-catenin signaling pathway strongly correlates with tumorigenesis and malignant progression in a number of cancers, including colorectal, breast, lung, glioblastoma, prostate, melanoma, ovarian and gastric [217, 219]. Recently, a low abundance transcriptome analysis was performed for GC, and the Wnt/Hh pathway was found to be deregulated. Furthermore, the genes that exhibited the largest differential expression in the GC samples analyzed included: WISP1, FZD5 and its ligand WNT5, CTBP1, PTCH, and SFRP4 [220]. Moreover, increased expression of *WNT5A* (which leads to β-catenin -independent signaling) has been found to potentially correlate with poor clinical outcome for cases of melanoma and GC [217].

Despite the fact that the Wnt signaling pathway is more difficult to target compared with the Notch and Hh pathways, receptor/ligand interactions, cytosolic signaling components, and nuclear signaling components of the Wnt signaling pathway have been inhibited [221]. Furthermore, these inhibitors can be grouped into two classes, small-molecule inhibitors (e.g., nonsteroidal anti-inflammatory drugs (NSAIDs), the CBP/β-catenin antagonist, ICG-001) and biologic inhibitors (antibodies, RNA interference (RNAi), and recombinant proteins) [216]. The majority of these inhibitors are in the preclinical stages of development, except PRI-724 which has been evaluated in clinical trials for colorectal and pancreatic patients [222].

The Wnt/β-catenin signaling pathway also contributes to the regulation of CSCs [223, 224]. In the past few years, numerous groups have worked to isolate and characterize GC stem cells [183, 225–228]. Li et al. [188] showed that stem cell-like circulating tumor cells are associated with a poor prognosis in GC. Several groups are also trying to understand how intrinsic and extrinsic factors that regulate Wnt/β-catenin signaling coordinate with CSC states in different malignancies, including GC [219, 229–234]. Mao et al. [219] have recently reported that activation of Wnt1 signaling accelerates the proliferation of gastric CSCs, whereas salinomycin, the first agent identified as a selective inhibitor of breast CSCs, acts to inhibit gastric tumor growth by suppressing Wnt signaling in CSCs both *in vitro* and *in vivo*.

## THE NOTCH SIGNALING PATHWAY

The Notch signaling pathway affects cell proliferation, differentiation, apoptosis, and stem cell maintenance [235] via four Notch receptors (NOTCH1/2/3/4) that each has extracellular and transmembrane domains. The corresponding family of Notch ligands is comprised of JAG1/2 and DLL1/3/4 (DLL1/3/4). When these ligands bind the EGF-like repeat regions in the Notch receptors, a metalloprotease of the ADAM family executes a cleavage reaction [235]. Following the execution of a second intramembrane cleavage event by an γ-secretase complex, the intracellular domain of the Notch receptor is released (NotchIC or NICD). Upon translocation of this domain into the nucleus [236], its works as a transcriptional coactivator, thus regulating the expression of several target genes [237].

The Notch signaling pathway is involved in normal gastric mucosa development and mediates the differentiation of the gastric epithelium into foveolar glands [238]. However, higher levels of Notch receptors (e.g., NOTCH1/2/3) and Notch ligands (e.g., JAG1/2) have been detected in samples of gastric premalignant lesions and GC tissues [238, 239]. In a recent meta-analysis, NOTCH1 expression was found to be significantly higher in GC tissues than in normal tissues, especially in samples of intestinal metaplasia and well-differentiated intestinal-type GC, thereby suggesting a crucial role for NOTCH1 in both promoting the metaplastic transition and maintaining the proliferation of intestinalized cells [240]. The activated form of the NOTCH1 receptor has also been found to promote the formation of colonies and xenografted tumor growth of human stomach adenocarcinoma SC-M1 cells [240]. Furthermore, positive expression of Notch1 or Jagged1 protein has been proven to be associated with a poor prognosis and both have been found to be independent prognostic predictors in GC [240, 241]. These results are consistent with a role for *NOTCH1* as an oncogene in many solid malignancies. To date, mutated *NOTCH1* has only been detected in T-cell acute lymphoblastic leukemia, and not in other common human cancers, including GC [242]. Interestingly, GC patients with a larger tumor ( > 5 cm), positive lymphovascular invasion, and distal metastasis had significantly higher expression rates of NOTCH1, thereby suggesting that NOTCH1 may also participate in tumor progression and metastasis of GC [240].

When JAG1 expression was compared between GC tissues and normal tissues, no significant difference was observed. In contrast, expression levels of DLL4 and HES1 have been found to be significantly higher in GC tissues than in normal tissues, with the levels of DLL4 being overexpressed in advanced stage GC patients. Levels of NOTCH2 and NOTCH3 expression have also exhibited significant overexpression in GC tissues compared to normal tissues. However, while no difference in NOTCH2 expression levels have been observed between intestinal-type and diffuse-type cancers, levels of NOTCH3 and JAG2 are significantly higher in the intestinal-type group. It has been demonstrated that activation of Notch2 signaling promotes cell proliferation and xenograft tumor [240].

Notch receptor cleavage can be disrupted by γ-secretase inhibitors (GSIs) such as: L685, RO4929097, PF-03084014, and DAPT. Correspondingly, GSIs have been shown to inhibit cell growth and to induce apoptosis in hepatoma, breast, pancreatic, and myeloma cancers [243–245]. Given that the Notch signaling pathway participates in many processes of cellular physiology, it has been hypothesized that inactivation of γ-secretases can lead to pathological dysfunction of various tissues and organs. However, GSIs do not only target Notch signaling proteins. GSIs also target proteases, and therefore, may have widespread adverse effects *in vivo* [245, 246]. In GC cells, inhibition of the NOTCH2 pathway by γ-secretase antagonists did not lead to growth arrest or cell death [238]. However, this may be due to compensation from other signaling pathways in response to suppressed Notch signaling activity [238].

Currently, there are two γ-secretases that are being evaluated in phase I clinical trials. RO4929097 is being evaluated in patients with breast cancer, pancreatic cancer, leukemia, sarcoma, melanoma, or other solid tumors (NCT01131234), whereas PF-03084014 is being tested in patients with advances cancer and leukemia (NCT00878189) [92].

## THE HIPPO SIGNALING PATHWAY

In mammals, the Hippo signaling pathway provides tumor-suppressor signaling involved in regulation of diverse cellular processes such as proliferation, apoptosis, survival, migration and differentiation [247]. In recent years, growing evidence has pointed towards an oncogenic role for the Hippo signaling pathway in human cancer, including GC. While the key components and upstream regulators of the Hippo signaling pathway (MST1/2, LATS1, SAV1, MOB, FAT, NF2, and FDM6) are mostly considered to participate as tumor suppressors, downstream mediators in this pathway (TAZ, YAP1, and TEAD) are mostly involved in oncogenic events. The first report of Hippo pathway deregulation in gastric epithelial tumorigenesis was described by Lam-Himlin et al. [248], where YAP1 expression in the cytoplasm and nucleus were found to significantly increase in high-grade dysplasia, adenocarcinoma, and metastasis gastric disease. Positive expression of YAP1 was also detected in 79.2% of GCs, 47.1% of dysplasia cases, and 15.0% of normal gastric tissues [249]. Nuclear overexpression of YAP1 has been described as an independent biomarker for poor survival, especially in patients with early stage GC [249, 250]. Additionally, Xu et al. [251] showed that expression of MST1/2 and LATS1, two principal suppressor genes of this pathway, were down-regulated in GC compared with normal gastric epithelium and adenoma. Moreover, the expression levels of SAV1 and LATS1 in GC patients with lymph node metastasis were significantly lower than those in GC patients without lymph node metastasis. Knockdown of YAP1 has also resulted in a significant reduction in cell proliferation, anchorage-dependent colony formation, cell invasion, and cell motility. Meanwhile, ectopic YAP1 expression induced a more invasive phenotype and accelerated cell growth in both *in vitro* and *in vivo* assays [249]. Taken together, these findings suggest that inhibition of YAP1 may represent a prognostic biomarker and a potential therapeutic target for GC.

The identification of small molecule inhibitors of YAP1 is also an active area of study. In a screening of more than 3300 drugs by Liu-Chittenden et al. [252], the porphyrin family of proteins, including verteporfin (VP), hematoporphyrin, and protoporphyrin IX, were identified as YAP1 inhibitors. When livers were treated with VP, overgrowth induced by YAP overexpression, or by inactivation of NF2, was inhibited [252, 253], thereby demonstrating the therapeutic potential of disrupting YAP1/TAZ-TEAD interactions. In a recent phase I/II study, the feasibility and safety of using VP photodynamic therapy for the treatment of locally advanced stage pancreatic cancer was demonstrated [254]. However, despite these promising data, it remains to be determined whether inhibitors of YAP1 will be effective for the treatment of GC.

## CONCLUDING REMARKS

Emerging data from high-throughput technologies have provided valuable insight into the molecular classification and intracellular pathways that are relevant to GC, and this facilitates the development of novel strategies for treating GC. However, chemotherapy resistance remains a significant challenge in treating GC due to the heterogeneity of these tumors and their CSC component that lead to high rates of recurrence. New strategies should seek to intervene in cancer or tissue-specific targets, enabling the protection of normal cells as well [85]. Thus, there is a critical need for therapies that are based on genetic and epigenetic aberrations that modulate different signaling pathways, or combinations of different signaling pathways, in various types of cancer, particularly GC. As a result, the development of more effective agents and the identification biomarkers that can be used for the diagnosis, prognosis, and therapy of patients who might benefit from specific targeted therapies can be elucidated (Figure 3).

Despite the ongoing development of drugs for the treatment of unresectable or metastatic gastric adenocarcinoma, the overall prognosis for these diseases remains poor. Moreover, many of these strategies are generally only applicable to a limited number of patients. To date, advances in personalized medicine have improved therapeutic responses in advanced stage ERBB2-positive GC patients treated with trastuzumab. However, this therapy has benefited only ~15% of these patients. New molecules that target the VEGF, PI3K/AKT/mTOR, and MET signaling pathways are also under investigation, and promising results have been obtained. Novel insights regarding signaling pathways that regulate gastric CSCs such as Hh, Notch, and Hippo, and the drugs that block these pathways, also have the potential to improve treatment responses to targeted therapy alone, or in combination with conventional cytotoxic therapy. Therefore, a new paradigm in GC research that involves the implementation of strategies and clinical trials in which patients can be classified based on molecular characteristics or molecular subtypes in order to select the most appropriate and target-specific therapies for patients with advanced stage GC is recommended.

## References

[1] Jemal A, Bray F, Center MM, Ferlay J, Ward E, Forman D (2011). Global cancer statistics. CA Cancer J Clin.

[2] Nadauld LD, Ford JM (2013). Molecular profiling of gastric cancer: toward personalized cancer medicine. J Clin Oncol.

[3] Lee HS, Cho SB, Lee HE, Kim MA, Kim JH, Park do J, Kim JH, Yang HK, Lee BL, Kim WH (2007). Protein expression profiling and molecular classification of gastric cancer by the tissue array method. Clin Cancer Res.

[4] The Cancer Genome Atlas Research N (2014). Comprehensive molecular characterization of gastric adenocarcinoma. Nature.

[5] Razzak M (2014). Genetics: new molecular classification of gastric adenocarcinoma proposed by The Cancer Genome Atlas. Nat Rev Clin Oncol.

[6] Stock M, Otto F (2005). Gene deregulation in gastric cancer. Gene.

[7] Rivera F, Gravalos C, Garcia-Carbonero R, Seom (2012). SEOM clinical guidelines for the diagnosis and treatment of gastric adenocarcinoma. Clin Transl Oncol.

[8] Dicken BJ, Bigam DL, Cass C, Mackey JR, Joy AA, Hamilton SM (2005). Gastric adenocarcinoma: review and considerations for future directions. Ann Surg.

[9] Macdonald JS, Smalley SR, Benedetti J, Hundahl SA, Estes NC, Stemmermann GN, Haller DG, Ajani JA, Gunderson LL, Jessup JM, Martenson JA (2001). Chemoradiotherapy after surgery compared with surgery alone for adenocarcinoma of the stomach or gastroesophageal junction. N Engl J Med.

[10] Goodman KA (2015). Refining the Role for Adjuvant Radiotherapy in Gastric Cancer: Risk Stratification Is Key. J Clin Oncol.

[11] Lee J, Lim do H, Kim S, Park SH, Park JO, Park YS, Lim HY, Choi MG, Sohn TS, Noh JH, Bae JM, Ahn YC, Sohn I (2012). Phase III trial comparing capecitabine plus cisplatin versus capecitabine plus cisplatin with concurrent capecitabine radiotherapy in completely resected gastric cancer with D2 lymph node dissection: the ARTIST trial. J Clin Oncol.

[12] Cunningham D, Allum WH, Stenning SP, Thompson JN, Van de Velde CJ, Nicolson M, Scarffe JH, Lofts FJ, Falk SJ, Iveson TJ, Smith DB, Langley RE, Verma M, Weeden S, Chua YJ, Participants MT (2006). Perioperative chemotherapy versus surgery alone for resectable gastroesophageal cancer. N Engl J Med.

[13] Paoletti X, Oba K, Burzykowski T, Michiels S, Ohashi Y, Pignon JP, Rougier P, Sakamoto J, Sargent D, Sasako M, Van Cutsem E, Buyse M (2010). Benefit of adjuvant chemotherapy for resectable gastric cancer: a meta-analysis. JAMA.

[14] Sakuramoto S, Sasako M, Yamaguchi T, Kinoshita T, Fujii M, Nashimoto A, Furukawa H, Nakajima T, Ohashi Y, Imamura H, Higashino M, Yamamura Y, Kurita A, Arai K, Group A-G (2007). Adjuvant chemotherapy for gastric cancer with S-1, an oral fluoropyrimidine. N Engl J Med.

[15] Noh SH, Park SR, Yang HK, Chung HC, Chung IJ, Kim SW, Kim HH, Choi JH, Kim HK, Yu W, Lee JI, Shin DB, Ji J (2014). Adjuvant capecitabine plus oxaliplatin for gastric cancer after D2 gastrectomy (CLASSIC): 5-year follow-up of an open-label, randomised phase 3 trial. Lancet Oncol.

[16] Wagner AD, Unverzagt S, Grothe W, Kleber G, Grothey A, Haerting J, Fleig WE (2010). Chemotherapy for advanced gastric cancer. Cochrane Database Syst Rev.

[17] Garrido M, Fonseca PJ, Vieitez JM, Frunza M, Lacave AJ (2014). Challenges in first line chemotherapy and targeted therapy in advanced gastric cancer. Expert Rev Anticancer Ther.

[18] Kang Y, Kang W, Shin D, Chen J, Xiong J, Wang J, Lichinitser M, Guan Z, Khasanov R, Zheng L, Philco-Salas M, Suarez T, Santamaria J, Forster G, McCloud P (2009). Capecitabine/cisplatin versus 5-fluorouracil/cisplatin as first-line therapy in patients with advanced gastric cancer: a randomised phase III noninferiority trial. Ann Oncol.

[19] Koizumi W, Narahara H, Hara T, Takagane A, Akiya T, Takagi M, Miyashita K, Nishizaki T, Kobayashi O, Takiyama W, Toh Y, Nagaie T, Takagi S (2008). S-1 plus cisplatin versus S-1 alone for first-line treatment of advanced gastric cancer (SPIRITS trial): a phase III trial. Lancet Oncol.

[20] Yamada Y, Higuchi K, Nishikawa K, Gotoh M, Fuse N, Sugimoto N, Nishina T, Amagai K, Chin K, Niwa Y, Tsuji A, Imamura H, Tsuda M (2015). Phase III study comparing oxaliplatin plus S-1 with cisplatin plus S-1 in chemotherapy-naive patients with advanced gastric cancer. Ann Oncol.

[21] Al-Batran SE, Hartmann JT, Probst S, Schmalenberg H, Hollerbach S, Hofheinz R, Rethwisch V, Seipelt G, Homann N, Wilhelm G, Schuch G, Stoehlmacher J, Derigs HG (2008). Phase III trial in metastatic gastroesophageal adenocarcinoma with fluorouracil, leucovorin plus either oxaliplatin or cisplatin: a study of the Arbeitsgemeinschaft Internistische Onkologie. J Clin Oncol.

[22] Cunningham D, Starling N, Rao S, Iveson T, Nicolson M, Coxon F, Middleton G, Daniel F, Oates J, Norman AR, Upper Gastrointestinal Clinical Studies Group of the National Cancer Research Institute of the United K (2008). Capecitabine and oxaliplatin for advanced esophagogastric cancer. N Engl J Med.

[23] Ford HE, Marshall A, Bridgewater JA, Janowitz T, Coxon FY, Wadsley J, Mansoor W, Fyfe D, Madhusudan S, Middleton GW, Swinson D, Falk S, Chau I (2014). Docetaxel versus active symptom control for refractory oesophagogastric adenocarcinoma (COUGAR-02): an open-label, phase 3 randomised controlled trial. Lancet Oncol.

[24] Ajani JA, Moiseyenko VM, Tjulandin S, Majlis A, Constenla M, Boni C, Rodrigues A, Fodor M, Chao Y, Voznyi E, Marabotti C, Van Cutsem E, Group VS (2007). Clinical benefit with docetaxel plus fluorouracil and cisplatin compared with cisplatin and fluorouracil in a phase III trial of advanced gastric or gastroesophageal cancer adenocarcinoma: the V-325 Study Group. J Clin Oncol.

[25] Dank M, Zaluski J, Barone C, Valvere V, Yalcin S, Peschel C, Wenczl M, Goker E, Cisar L, Wang K, Bugat R (2008). Randomized phase III study comparing irinotecan combined with 5-fluorouracil and folinic acid to cisplatin combined with 5-fluorouracil in chemotherapy naive patients with advanced adenocarcinoma of the stomach or esophagogastric junction. Ann Oncol.

[26] Hironaka S, Ueda S, Yasui H, Nishina T, Tsuda M, Tsumura T, Sugimoto N, Shimodaira H, Tokunaga S, Moriwaki T, Esaki T, Nagase M, Fujitani K (2013). Randomized, open-label, phase III study comparing irinotecan with paclitaxel in patients with advanced gastric cancer without severe peritoneal metastasis after failure of prior combination chemotherapy using fluoropyrimidine plus platinum: WJOG 4007 trial. J Clin Oncol.

[27] Bang YJ, Van Cutsem E, Feyereislova A, Chung HC, Shen L, Sawaki A, Lordick F, Ohtsu A, Omuro Y, Satoh T, Aprile G, Kulikov E, Hill J (2010). Trastuzumab in combination with chemotherapy versus chemotherapy alone for treatment of HER2-positive advanced gastric or gastro-oesophageal junction cancer (ToGA): a phase 3, open-label, randomised controlled trial. Lancet.

[28] Kim JW, Im SA, Kim M, Cha Y, Lee KH, Keam B, Kim MA, Han SW, Oh DY, Kim TY, Kim WH, Bang YJ (2012). The prognostic significance of HER2 positivity for advanced gastric cancer patients undergoing first-line modified FOLFOX-6 regimen. Anticancer Res.

[29] Kim MA, Lee HS, Lee HE, Jeon YK, Yang HK, Kim WH (2008). EGFR in gastric carcinomas: prognostic significance of protein overexpression and high gene copy number. Histopathology.

[30] Terashima M, Kitada K, Ochiai A, Ichikawa W, Kurahashi I, Sakuramoto S, Katai H, Sano T, Imamura H, Sasako M, ACTS-GC Group (2012). Impact of expression of human epidermal growth factor receptors EGFR and ERBB2 on survival in stage II/III gastric cancer. Clin Cancer Res.

[31] Gravalos C, Gomez-Martin C, Rivera F, Ales I, Queralt B, Marquez A, Jimenez U, Alonso V, Garcia-Carbonero R, Sastre J, Colomer R, Cortes-Funes H, Jimeno A (2011). Phase II study of trastuzumab and cisplatin as first-line therapy in patients with HER2-positive advanced gastric or gastroesophageal junction cancer. Clin Transl Oncol.

[32] Lauren P (1965). The Two Histological Main Types of Gastric Carcinoma: Diffuse and So-Called Intestinal-Type Carcinoma. An Attempt at a Histo-Clinical Classification. Acta Pathol Microbiol Scand.

[33] Correa P (1992). Human Gastric Carcinogenesis: A Multistep and Multifactorial Process—First American Cancer Society Award Lecture on Cancer Epidemiology and Prevention. Cancer Res.

[34] Peek RM, Crabtree JE (2006). Helicobacter infection and gastric neoplasia. J Pathol.

[35] Gomceli I, Demiriz B, Tez M (2012). Gastric carcinogenesis. World J Gastroenterol.

[36] Yasui W, Oue N, Kuniyasu H, Ito R, Tahara E, Yokozaki H (2001). Molecular diagnosis of gastric cancer: present and future. Gastric Cancer.

[37] Tahara E (2004). Genetic pathways of two types of gastric cancer. IARC Sci Publ.

[38] Zang ZJ, Cutcutache I, Poon SL, Zhang SL, McPherson JR, Tao J, Rajasegaran V, Heng HL, Deng N, Gan A, Lim KH, Ong CK, Huang D (2012). Exome sequencing of gastric adenocarcinoma identifies recurrent somatic mutations in cell adhesion and chromatin remodeling genes. Nat Genet.

[39] Lee YS, Cho YS, Lee GK, Lee S, Kim YW, Jho S, Kim HM, Hong SH, Hwang JA, Kim SY, Hong D, Choi IJ, Kim BC (2014). Genomic profile analysis of diffuse-type gastric cancers. Genome Biol.

[40] Kakiuchi M, Nishizawa T, Ueda H, Gotoh K, Tanaka A, Hayashi A, Yamamoto S, Tatsuno K, Katoh H, Watanabe Y, Ichimura T, Ushiku T, Funahashi S (2014). Recurrent gain-of-function mutations of RHOA in diffuse-type gastric carcinoma. Nat Genet.

[41] Meining A, Morgner A, Miehlke S, Bayerdorffer E, Stolte M (2001). Atrophy-metaplasia-dysplasia-carcinoma sequence in the stomach: a reality or merely an hypothesis?. Best Pract Res Clin Gastroenterol.

[42] Tang W, Morgan DR, Meyers MO, Dominguez RL, Martinez E, Kakudo K, Kuan PF, Banet N, Muallem H, Woodward K, Speck O, Gulley ML (2012). Epstein-barr virus infected gastric adenocarcinoma expresses latent and lytic viral transcripts and has a distinct human gene expression profile. Infect Agent Cancer.

[43] Khan G, Hashim MJ (2014). Global burden of deaths from Epstein-Barr virus attributable malignancies 1990-2010. Infect Agent Cancer.

[44] Iizasa H, Nanbo A, Nishikawa J, Jinushi M, Yoshiyama H (2012). Epstein-Barr Virus (EBV)-associated Gastric Carcinoma. Viruses.

[45] Akiba S, Koriyama C, Herrera-Goepfert R, Eizuru Y (2008). Epstein-Barr virus associated gastric carcinoma: epidemiological and clinicopathological features. Cancer Sci.

[46] Camargo MC, Kim WH, Chiaravalli AM, Kim KM, Corvalan AH, Matsuo K, Yu J, Sung JJ, Herrera-Goepfert R, Meneses-Gonzalez F, Kijima Y, Natsugoe S, Liao LM (2014). Improved survival of gastric cancer with tumour Epstein-Barr virus positivity: an international pooled analysis. Gut.

[47] Arcaro A, Guerreiro AS (2007). The phosphoinositide 3-kinase pathway in human cancer: genetic alterations and therapeutic implications. Curr Genomics.

[48] Hoadley KA, Yau C, Wolf DM, Cherniack AD, Tamborero D, Ng S, Leiserson MD, Niu B, McLellan MD, Uzunangelov V, Zhang J, Kandoth C, Akbani R (2014). Multiplatform analysis of 12 cancer types reveals molecular classification within and across tissues of origin. Cell.

[49] Kandoth C, McLellan MD, Vandin F, Ye K, Niu B, Lu C, Xie M, Zhang Q, McMichael JF, Wyczalkowski MA, Leiserson MD, Miller CA, Welch JS (2013). Mutational landscape and significance across 12 major cancer types. Nature.

[50] Wu H, Huang M, Cao P, Wang T, Shu Y, Liu P (2012). MiR-135a targets JAK2 and inhibits gastric cancer cell proliferation. Cancer Biol Ther.

[51] Sun J, Xu K, Wu C, Wang Y, Hu Y, Zhu Y, Chen Y, Shi Q, Yu G, Zhang X (2007). PD-L1 expression analysis in gastric carcinoma tissue and blocking of tumor-associated PD-L1 signaling by two functional monoclonal antibodies. Tissue Antigens.

[52] McDermott D, Atkins M (2013). PD-1 as a potential target in cancer therapy. Cancer Med.

[53] Kim JY, Shin NR, Kim A, Lee HJ, Park WY, Kim JY, Lee CH, Huh GY, Park do Y (2013). Microsatellite instability status in gastric cancer: a reappraisal of its clinical significance and relationship with mucin phenotypes. Korean J Pathol.

[54] Choi YY, Bae JM, An JY, Kwon IG, Cho I, Shin HB, Eiji T, Aburahmah M, Kim HI, Cheong JH, Hyung WJ, Noh SH (2014). Is microsatellite instability a prognostic marker in gastric cancer? A systematic review with meta-analysis. J Surg Oncol.

[55] Falchetti M, Saieva C, Lupi R, Masala G, Rizzolo P, Zanna I, Ceccarelli K, Sera F, Mariani-Costantini R, Nesi G, Palli D, Ottini L (2008). Gastric cancer with high-level microsatellite instability: target gene mutations, clinicopathologic features, and long-term survival. Hum Pathol.

[56] Yamamoto H, Sawai H, Perucho M (1997). Frameshift somatic mutations in gastrointestinal cancer of the microsatellite mutator phenotype. Cancer Res.

[57] Gazvoda B, Juvan R, Zupanic-Pajnic I, Repse S, Ferlan-Marolt K, Balazic J, Komel R (2007). Genetic changes in Slovenian patients with gastric adenocarcinoma evaluated in terms of microsatellite DNA. Eur J Gastroenterol Hepatol.

[58] Karaman A, Kabalar ME, Binici DN, Ozturk C, Pirim I (2010). Genetic alterations in gastric precancerous lesions. Genet Couns.

[59] French AJ, Petroni G, Thibideau SN, Smolkin M, Bissonette E, Roviello F, Harper JC, Koch BR, Anderson SA, Hebbring SJ, Powell SM (2004). Allelic imbalance of 8p indicates poor survival in gastric cancer. J Mol Diagn.

[60] McLean MH, El-Omar EM (2014). Genetics of gastric cancer. Nat Rev Gastroenterol Hepatol.

[61] Wang K, Yuen ST, Xu JC, Lee SP, Yan HHN, Shi ST, Siu HC, Deng SB, Chu KM, Law S, Chan KH, Chan ASY, Tsui WY (2014). Whole-genome sequencing and comprehensive molecular profiling identify new driver mutations in gastric cancer. Nat Genet.

[62] Humar B, Guilford P (2009). Hereditary diffuse gastric cancer: a manifestation of lost cell polarity. Cancer Sci.

[63] Lin M-T, Lin B-R, Chang C-C, Chu C-Y, Su H-J, Chen S-T, Jeng Y-M, Kuo M-L (2007). IL-6 induces AGS gastric cancer cell invasion via activation of the c-Src/RhoA/ROCK signaling pathway. Int J Cancer.

[64] Murray D, Horgan G, MacMathuna P, Doran P (2008). NET1-mediated RhoA activation facilitates lysophosphatidic acid-induced cell migration and invasion in gastric cancer. Br J Cancer.

[65] Liu N, Bi F, Pan Y, Sun L, Xue Y, Shi Y, Yao X, Zheng Y, Fan D (2004). Reversal of the Malignant Phenotype of Gastric Cancer Cells by Inhibition of RhoA Expression and Activity. Clin Cancer Res.

[66] Ayed-Guerfali DB, Hassairi B, Khabir A, Sellami-Boudawara T, Gargouri A, Mokdad-Gargouri R (2014). Expression of APC, beta-catenin and E-cadherin in Tunisian patients with gastric adenocarcinoma: clinical significance. Tumour Biol.

[67] Deng N, Goh LK, Wang H, Das K, Tao J, Tan IB, Zhang S, Lee M, Wu J, Lim KH, Lei Z, Goh G, Lim QY (2012). A comprehensive survey of genomic alterations in gastric cancer reveals systematic patterns of molecular exclusivity and co-occurrence among distinct therapeutic targets. Gut.

[68] Lee SJ, Do IG, Lee J, Kim KM, Jang J, Sohn I, Kang WK (2013). Gastric cancer (GC) patients with hedgehog pathway activation: PTCH1 and GLI2 as independent prognostic factors. Target Oncol.

[69] Yang W, Raufi A, Klempner SJ (2014). Targeted therapy for gastric cancer: molecular pathways and ongoing investigations. Biochim Biophys Acta.

[70] Roskoski R (2014). The ErbB/HER family of protein-tyrosine kinases and cancer. Pharmacol Res.

[71] Dragovich T, McCoy S, Fenoglio-Preiser CM, Wang J, Benedetti JK, Baker AF, Hackett CB, Urba SG, Zaner KS, Blanke CD, Abbruzzese JL (2006). Phase II trial of erlotinib in gastroesophageal junction and gastric adenocarcinomas: SWOG 0127. J Clin Oncol.

[72] Janmaat ML, Gallegos-Ruiz MI, Rodriguez JA, Meijer GA, Vervenne WL, Richel DJ, Van Groeningen C, Giaccone G (2006). Predictive factors for outcome in a phase II study of gefitinib in second-line treatment of advanced esophageal cancer patients. J Clin Oncol.

[73] Ferry DR, Anderson M, Beddard K, Tomlinson S, Atherfold P, Obszynska J, Harrison R, Jankowski J (2007). A phase II study of gefitinib monotherapy in advanced esophageal adenocarcinoma: evidence of gene expression, cellular, and clinical response. Clin Cancer Res.

[74] Chan JA, Blaszkowsky LS, Enzinger PC, Ryan DP, Abrams TA, Zhu AX, Temel JS, Schrag D, Bhargava P, Meyerhardt JA, Wolpin BM, Fidias P, Zheng H, Florio S, Regan E, Fuchs CS (2011). A multicenter phase II trial of single-agent cetuximab in advanced esophageal and gastric adenocarcinoma. Ann Oncol.

[75] Pinto C, Di Fabio F, Siena S, Cascinu S, Rojas Llimpe FL, Ceccarelli C, Mutri V, Giannetta L, Giaquinta S, Funaioli C, Berardi R, Longobardi C, Piana E, Martoni AA (2007). Phase II study of cetuximab in combination with FOLFIRI in patients with untreated advanced gastric or gastroesophageal junction adenocarcinoma (FOLCETUX study). Ann Oncol.

[76] Han SW, Oh DY, Im SA, Park SR, Lee KW, Song HS, Lee NS, Lee KH, Choi IS, Lee MH, Kim MA, Kim WH, Bang YJ, Kim TY (2009). Phase II study and biomarker analysis of cetuximab combined with modified FOLFOX6 in advanced gastric cancer. Br J Cancer.

[77] Kim C, Lee JL, Ryu MH, Chang HM, Kim TW, Lim HY, Kang HJ, Park YS, Ryoo BY, Kang YK (2011). A prospective phase II study of cetuximab in combination with XELOX (capecitabine and oxaliplatin) in patients with metastatic and/or recurrent advanced gastric cancer. Invest New Drugs.

[78] Lordick F, Luber B, Lorenzen S, Hegewisch-Becker S, Folprecht G, Woll E, Decker T, Endlicher E, Rothling N, Schuster T, Keller G, Fend F, Peschel C (2010). Cetuximab plus oxaliplatin/leucovorin/5-fluorouracil in first-line metastatic gastric cancer: a phase II study of the Arbeitsgemeinschaft Internistische Onkologie (AIO). Br J Cancer.

[79] Lordick F, Kang YK, Chung HC, Salman P, Oh SC, Bodoky G, Kurteva G, Volovat C, Moiseyenko VM, Gorbunova V, Park JO, Sawaki A, Celik I (2013). Capecitabine and cisplatin with or without cetuximab for patients with previously untreated advanced gastric cancer (EXPAND): a randomised, open-label phase 3 trial. Lancet Oncol.

[80] Vanhoefer U, Tewes M, Rojo F, Dirsch O, Schleucher N, Rosen O, Tillner J, Kovar A, Braun AH, Trarbach T, Seeber S, Harstrick A, Baselga J (2004). Phase I study of the humanized antiepidermal growth factor receptor monoclonal antibody EMD72000 in patients with advanced solid tumors that express the epidermal growth factor receptor. J Clin Oncol.

[81] Trarbach T, Przyborek M, Schleucher N, Heeger S, Lupfert C, Vanhoefer U (2013). Phase I study of matuzumab in combination with 5-fluorouracil, leucovorin and cisplatin (PLF) in patients with advanced gastric and esophagogastric adenocarcinomas. Invest New Drugs.

[82] Sehrish R (2014). Gastric Cancer: The Ghosts of Past, Present and Future.

[83] Stahl P, Seeschaaf C, Lebok P, Kutup A, Bockhorn M, Izbicki JR, Bokemeyer C, Simon R, Sauter G, Marx AH (2015). Heterogeneity of amplification of HER2, EGFR, CCND1 and MYC in gastric cancer. BMC Gastroenterol.

[84] Blagosklonny MV (2004). How Cancer Could be Cured by 2015. Cell Cycle.

[85] Yamada Y (2013). Molecular therapy for gastric cancer. Chin Clin Oncol.

[86] Takahashi N, Yamada Y, Taniguchi H, Fukahori M, Sasaki Y, Shoji H, Honma Y, Iwasa S, Takashima A, Kato K, Hamaguchi T, Shimada Y (2014). Clinicopathological features and prognostic roles of KRAS, BRAF, PIK3CA and NRAS mutations in advanced gastric cancer. BMC Res Notes.

[87] Smyth EC, Cunningham D (2012). Targeted therapy for gastric cancer. Curr Treat Options Oncol.

[88] Vu T, Claret FX (2012). Trastuzumab: updated mechanisms of action and resistance in breast cancer. Front Oncol.

[89] Hudis CA (2007). Trastuzumab--mechanism of action and use in clinical practice. N Engl J Med.

[90] Kurokawa Y, Sugimoto N, Miwa H, Tsuda M, Nishina S, Okuda H, Imamura H, Gamoh M, Sakai D, Shimokawa T, Komatsu Y, Doki Y, Tsujinaka T, Furukawa H (2014). Phase II study of trastuzumab in combination with S-1 plus cisplatin in HER2-positive gastric cancer (HERBIS-1). Br J Cancer.

[91] ClinicalTrials.gov, U.S National Institutes of Health https://clinicaltrials.gov.

[92] Satoh T, Xu RH, Chung HC, Sun GP, Doi T, Xu JM, Tsuji A, Omuro Y, Li J, Wang JW, Miwa H, Qin SK, Chung IJ (2014). Lapatinib plus paclitaxel versus paclitaxel alone in the second-line treatment of HER2-amplified advanced gastric cancer in Asian populations: TyTAN--a randomized, phase III study. J Clin Oncol.

[93] Hecht JR, Bang YJ, Qin S, Chung HC, Xu J, Park JO, Jeziorski K, Shparyk Y, Hoff PM, Sobrero AF, Salman P, Li J, Protsenko S (2013). Lapatinib in combination with capecitabine plus oxaliplatin (CapeOx) in HER2-positive advanced or metastatic gastric, esophageal, or gastroesophageal adenocarcinoma (AC): The TRIO-013/LOGiC. J Clin Oncol.

[94] Bergers G, Benjamin LE (2003). Tumorigenesis and the angiogenic switch. Nat Rev Cancer.

[95] Zhou Y, Li G, Wu J, Zhang Z, Wu Z, Fan P, Hao T, Zhang X, Li M, Zhang F, Li Q, Lu B, Qiao L (2010). Clinicopathological significance of E-cadherin, VEGF, and MMPs in gastric cancer. Tumour Biol.

[96] Chen J, Zhou SJ, Zhang Y, Zhang GQ, Zha TZ, Feng YZ, Zhang K (2013). Clinicopathological and prognostic significance of galectin-1 and vascular endothelial growth factor expression in gastric cancer. World J Gastroenterol.

[97] Lee SJ, Kim JG, Sohn SK, Chae YS, Moon JH, Kim SN, Bae HI, Chung HY, Yu W (2009). No association of vascular endothelial growth factor-A (VEGF-A) and VEGF-C expression with survival in patients with gastric cancer. Cancer Res Treat.

[98] Deguchi K, Ichikawa D, Soga K, Watanabe K, Kosuga T, Takeshita H, Konishi H, Morimura R, Tsujiura M, Komatsu S, Shiozaki A, Okamoto K, Fujiwara H, Otsuji E (2010). Clinical significance of vascular endothelial growth factors C and D and chemokine receptor CCR7 in gastric cancer. Anticancer Res.

[99] Gou HF, Chen XC, Zhu J, Jiang M, Yang Y, Cao D, Hou M (2011). Expressions of COX-2 and VEGF-C in gastric cancer: correlations with lymphangiogenesis and prognostic implications. J Exp Clin Canc Res.

[100] Ohtsu A, Shah MA, Van Cutsem E, Rha SY, Sawaki A, Park SR, Lim HY, Yamada Y, Wu J, Langer B, Starnawski M, Kang YK (2011). Bevacizumab in combination with chemotherapy as first-line therapy in advanced gastric cancer: a randomized, double-blind, placebo-controlled phase III study. J Clin Oncol.

[101] Van Cutsem E, de Haas S, Kang YK, Ohtsu A, Tebbutt NC, Ming Xu J, Peng Yong W, Langer B, Delmar P, Scherer SJ, Shah MA (2012). Bevacizumab in combination with chemotherapy as first-line therapy in advanced gastric cancer: a biomarker evaluation from the AVAGAST randomized phase III trial. J Clin Oncol.

[102] Fuchs CS, Tomasek J, Yong CJ, Dumitru F, Passalacqua R, Goswami C, Safran H, dos Santos LV, Aprile G, Ferry DR, Melichar B, Tehfe M, Topuzov E (2014). Ramucirumab monotherapy for previously treated advanced gastric or gastro-oesophageal junction adenocarcinoma (REGARD): an international, randomised, multicentre, placebo-controlled, phase 3 trial. Lancet.

[103] Wilke H, Muro K, Van Cutsem E, Oh SC, Bodoky G, Shimada Y, Hironaka S, Sugimoto N, Lipatov O, Kim TY, Cunningham D, Rougier P, Komatsu Y (2014). Ramucirumab plus paclitaxel versus placebo plus paclitaxel in patients with previously treated advanced gastric or gastro-oesophageal junction adenocarcinoma (RAINBOW): a double-blind, randomised phase 3 trial. Lancet Oncol.

[104] Li J, Qin S, Xu J, Guo W, Xiong J, Bai Y, Sun G, Yang Y, Wang L, Xu N, Cheng Y, Wang Z, Zheng L (2013). Apatinib for chemotherapy-refractory advanced metastatic gastric cancer: results from a randomized, placebo-controlled, parallel-arm, phase II trial. J Clin Oncol.

[105] Sun W, Powell M, O'Dwyer PJ, Catalano P, Ansari RH, Benson AB (2010). Phase II study of sorafenib in combination with docetaxel and cisplatin in the treatment of metastatic or advanced gastric and gastroesophageal junction adenocarcinoma: ECOG 5203. J Clin Oncol.

[106] Engelman JA, Luo J, Cantley LC (2006). The evolution of phosphatidylinositol 3-kinases as regulators of growth and metabolism. Nat Rev Genet.

[107] Liu JF ZX, Chen JH, Yi G, Chen HG, Ba MC, Lin SQ, Qi YC (2010). Up-regulation of PIK3CA promotes metastasis in gastric carcinoma. World J Gastroenterol.

[108] Tapia O, Riquelme I, Leal P, Sandoval A, Aedo S, Weber H, Letelier P, Bellolio E, Villaseca M, Garcia P, Roa J (2014). The PI3K/AKT/mTOR pathway is activated in gastric cancer with potential prognostic and predictive significance. Virchows Archiv.

[109] Ye B, Jiang L, Xu H, Zhou D, Li Z (2012). Expression of PI3K/AKT pathway in gastric cancer and its blockade suppresses tumor growth and metastasis. Int J Immunopathol Pharmacol.

[110] Cinti C, Vindigni C, Zamparelli A, Sala D, Epistolato M, Marrelli D, Cevenini G, Tosi P (2008). Activated Akt as an indicator of prognosis in gastric cancer. Virchows Archiv.

[111] Sangawa A, Shintani M, Yamao N, Kamoshida S (2014). Phosphorylation status of Akt and caspase-9 in gastric and colorectal carcinomas. Int J Clin Exp Pathol.

[112] Welker ME, Kulik G (2013). Recent syntheses of PI3K/Akt/mTOR signaling pathway inhibitors. Bioorg Med Chem.

[113] Brana I, Siu L (2012). Clinical development of phosphatidylinositol 3-kinase inhibitors for cancer treatment. BMC Med.

[114] Bhattacharya B, Akram M, Balasubramanian I, Tam KKY, Koh KX, Yee MQ, Soong R (2012). Pharmacologic synergy between dual phosphoinositide-3-kinase and mammalian target of rapamycin inhibition and 5-Fluorouracil in PIK3CA mutant gastric cancer cells. Cancer Biol Ther.

[115] Mazzoletti M, Bortolin F, Brunelli L, Pastorelli R, Di Giandomenico S, Erba E, Ubezio P, Broggini M (2011). Combination of PI3K/mTOR Inhibitors: Antitumor Activity and Molecular Correlates. Cancer Res.

[116] She Q, Chandarlapaty S, Ye Q, Lobo J, Haskell K, Leander K, DeFeo-Jones D, Huber H, Rosen N (2008). Breast tumor vells with PI3K mutation or HER2 amplification are selectively addicted to Akt signaling. PLoS One.

[117] Li J, Davies B, Han S, Zhou M, Bai Y, Zhang J, Xu Y, Tang L, Wang H, Liu Y, Yin X, Ji Q, Yu D-H (2013). The AKT inhibitor AZD5363 is selectively active in PI3KCA mutant gastric cancer, and sensitizes a patient-derived gastric cancer xenograft model with PTEN loss to Taxotere. J Transl Med.

[118] Davies B, Greenwood H, Dudley P (2012). Preclinical pharmacology of AZD5363, an inhibitor of AKT: pharmacodynamics, antitumor activity, and correlation of monotherapy activity with genetic background. Mol Canc Ther.

[119] Liu Y, Sun S, Li J, Yu D (2014). Targeting the PI3K/AKT Pathway for the Treatment of Gastric Cancer. Chemotherapy.

[120] Lin J, Sampath D, Nannini M (2013). Targeting activated Akt with GDC-0068, a novel selective Akt inhibitor that is efficacious in multiple tumor models. Clin Canc Res.

[121] Ma WW, Adjei AA (2009). Novel Agents on the Horizon for Cancer Therapy. CA Cancer J Clin.

[122] Dong M, Phan AT, Yao JC (2012). New Strategies for Advanced Neuroendocrine Tumors in the Era of Targeted Therapy. Clin Cancer Res.

[123] Lang S, Gaumann A, Koehl G (2007). Mammalian target of rapamycin is activated in human gastric cancer and serves as a target for therapy in an experimental model. Int J Cancer.

[124] Sun D, Zhang Y, Tian X, Chen Y, Fang J (2014). Inhibition of mTOR signalling potentiates the effects of trichostatin A in human gastric cancer cell lines by promoting histone acetylation. Cell Biol Int.

[125] Yoon D, Ryu M, Park Y (2012). Phase II study of everolimus with biomarker exploration in patients with advanced gastric cancer refractory to chemotherapy including fluoropyrimidine and platinum. Br J Cancer.

[126] Ohtsu A, Ajani JA, Bai Y-X, Bang Y-J, Chung H-C, Pan H-M, Sahmoud T, Shen L, Yeh K-H, Chin K, Muro K, Kim YH, Ferry D (2013). Everolimus for Previously Treated Advanced Gastric Cancer: Results of the Randomized, Double-Blind, Phase III GRANITE-1 Study. J Clin Oncol.

[127] Carracedo A, Ma L, Teruya-Feldstein J, Rojo F, Salmena L, Alimonti A, Egia A, Sasaki AT, Thomas G, Kozma SC, Papa A, Nardella C, Cantley LC, Baselga J, Pandolfi PP (2008). Inhibition of mTORC1 leads to MAPK pathway activation through a PI3K-dependent feedback loop in human cancer. J Clin Invest.

[128] Wong H, Yau T (2013). Molecular targeted therapies in advanced gastric cancer: does tumor histology matter?. Therap Adv Gastroenterol.

[129] Janku F, Tsimberidou AM, Garrido-Laguna I, Wang X, Luthra R, Hong DS, Naing A, Falchook GS, Moroney JW, Piha-Paul SA, Wheler JJ, Moulder SL, Fu S, Kurzrock R (2011). PIK3CA mutations in patients with advanced cancers treated with PI3K/AKT/mTOR axis inhibitors. Mol Cancer Ther.

[130] Park JH, Ryu MH, Park YS, Park SR, Na YS, Rhoo BY, Kang YK (2015). Successful control of heavily pretreated metastatic gastric cancer with the mTOR inhibitor everolimus (RAD001) in a patient with PIK3CA mutation and pS6 overexpression. BMC Cancer.

[131] Li V, Wong C, Chan T (2005). Mutations of PIK3CA in gastric adenocarcinoma. BMC Cancer.

[132] Oki E (2005). AKT phosphorylation associates with LOH of PTEN and leads to chemoresistance for gastric cancer. Int J Cancer.

[133] Shin J, Kim J, Lee S, Chae H, Kang J (2010). LY294002 may overcome 5-FU resistance via down-regulation of activated p-AKT in Epstein-Barr virus-positive gastric cancer cells. BMC Cancer.

[134] Oki E, Kakeji Y, Tokunaga E (2006). Impact of PTEN/AKT/PI3K signal pathway on the chemotherapy for gastric cancer. J Clin Oncol.

[135] Yu H-G, Ai Y-W, Yu L-L, Zhou X-D, Liu J, Li J-H, Xu X-M, Liu S, Chen J, Liu F, Qi Y-L, Deng Q, Cao J, Liu S-Q, Luo H-S, Yu J-P (2008). Phosphoinositide 3-kinase/Akt pathway plays an important role in chemoresistance of gastric cancer cells against etoposide and doxorubicin induced cell death. Int J Cancer.

[136] Im S, Lee K, Nam E (2005). Tumori.

[137] Sierra JR, Tsao M-S (2011). c-MET as a potential therapeutic target and biomarker in cancer. Ther Adv Med Oncol.

[138] Sharma N, Adjei AA (2011). In the clinic: ongoing clinical trials evaluating c-MET-inhibiting drugs. Ther Adv Med Oncol.

[139] Comoglio PM, Giordano S, Trusolino L (2008). Drug development of MET inhibitors: targeting oncogene addiction and expedience. Nat Rev Drug Discov.

[140] Ishibe S, Karihaloo A, Ma H, Zhang J, Marlier A, Mitobe M, Togawa A, Schmitt R, Czyczk J, Kashgarian M, Geller DS, Thorgeirsson SS, Cantley LG (2009). Met and the epidermal growth factor receptor act cooperatively to regulate final nephron number and maintain collecting duct morphology. Development.

[141] Lai AZ, Abella JV, Park M (2009). Crosstalk in Met receptor oncogenesis. Trends Cell Biol.

[142] Wu C, Li A, Chi C, Chung W, Liu T, Lui W, P'eng F (1998). Hepatocyte growth factor and Met/HGF receptors in patients with gastric adenocarcinoma. Oncol Rep.

[143] Amemiya H, Kono K, Itakura J, Feng Tang R, Takahashi A, An FQ, Kamei S, Iizuka H, Fujii H, Matsumoto Y (2002). c-Met Expression in Gastric Cancer with Liver Metastasis. Oncology.

[144] Taniguchi K, Yonemura Y, Nojima N, Hirono Y, Fushida S, Fujimura T, Miwa K, Endo Y, Yamamoto H, Watanabe H (1998). The relation between the growth patterns of gastric carcinoma and the expression of hepatocyte growth factor receptor (c-met), autocrine motility factor receptor, and urokinase-type plasminogen activator receptor. Cancer.

[145] Nakajima M, Sawada H, Yamada Y, Watanabe A, Tatsumi M, Yamashita J, Matsuda M, Sakaguchi T, Hirao T, Nakano H (1999). The prognostic significance of amplification and overexpression of c-met and c-erb B-2 in human gastric carcinomas. Cancer.

[146] Li Y, Chen C, He Y, Cai S, Yang D, He W, Xu J, Zan W (2012). Abnormal expression of E-cadherin in tumor cells is associated with poor prognosis of gastric carcinoma. J Surg Oncol.

[147] Kubicka S, Claas C, Staab S, Kühnel F, Zender L, Trautwein C, Wagner S, Rudolph KL, Manns M (2002). p53 mutation pattern and expression of c-erbB2 and c-met in gastric cancer: relation to histological subtypes, Helicobacter pylori infection, and prognosis. Dig Dis Sci.

[148] Huang TJ, Wang JY, Lin SR, Lian ST, Hsieh JS (2001). Overexpression of the c-met protooncogene in human gastric carcinoma--correlation to clinical features. Acta Oncol.

[149] Catenacci DVT, Cervantes G, Yala S, Nelson EA, El-Hashani E, Kanteti R, El Dinali M, Hasina R, Brägelmann J, Seiwert T, Sanicola M, Henderson L, Grushko TA (2011). RON (MST1R) is a novel prognostic marker and therapeutic target for gastroesophageal adenocarcinoma. Cancer Biol Ther.

[150] Zhao J, Zhang X, Xin Y (2011). Up-regulated expression of Ezrin and c-Met proteins are related to the metastasis and prognosis of gastric carcinomas. Histol Histopathol.

[151] Drebber U, Baldus SE, Nolden B, Grass G, Bollschweiler E, Dienes HP, Holscher AH, Monig SP (2008). The overexpression of c-met as a prognostic indicator for gastric carcinoma compared to p53 and p21 nuclear accumulation. Oncol Rep.

[152] Kawakami H, Okamoto I, Arao T, Okamoto W, Matsumoto K, Taniguchi H, Kuwata K, Yamaguchi H, Nishio K, Nakagawa K, Yamada Y (2013). MET amplification as a potential therapeutic target in gastric cancer. Oncotarget.

[153] Hack SP, Bruey JM, Koeppen H (2014). HGF/MET-directed therapeutics in gastroesophageal cancer: a review of clinical and biomarker development. Oncotarget.

[154] Iveson T, Donehower RC, Davidenko I, Tjulandin S, Deptala A, Harrison M, Nirni S, Lakshmaiah K, Thomas A, Jiang Y, Zhu M, Tang R, Anderson A, Dubey S, Oliner KS, Loh E (2014). Rilotumumab in combination with epirubicin, cisplatin, and capecitabine as first-line treatment for gastric or oesophagogastric junction adenocarcinoma: an open-label, dose de-escalation phase 1b study and a double-blind, randomised phase 2 study. Lancet Oncol.

[155] Merchant M, Ma X, Maun H (2013). Monovalent antibody design and mechanism of action of onartuzumab, a MET antagonist with anti-tumor activity as a therapeutic agent. Proc Natl Acad Sci USA.

[156] Martens T, Schmidt N-O, Eckerich C, Fillbrandt R, Merchant M, Schwall R, Westphal M, Lamszus K (2006). A Novel One-Armed Anti-c-Met Antibody Inhibits Glioblastoma Growth In vivo. Clin Cancer Res.

[157] Salgia R, Patel P, Bothos J, Yu W, Eppler S, Hegde P, Bai S, Kaur S, Nijem I, Catenacci D, Peterson A, Ratain M, Polite B, Mehnert J, Moss R (2014). Phase I Dose-Escalation Study of Onartuzumab as a Single Agent and in Combination with Bevacizumab in Patients with Advanced Solid Malignancies. Clin Cancer Res.

[158] Strickler JH, LoRusso P, Yen CJ, Lin CC, Kang YK, Kaminker P, Ansell P, Bhathena A, Wong S, Dudley MW, Naumovski L, Ramanathan RK (2014). Phase 1, open-label, dose-escalation, and expansion study of ABT-700, an anti-C-met antibody, in patients (pts) with advanced solid tumors. J Clin Oncol.

[159] Kang YK, LoRusso P, Salgia R, Yen CJ, Ramanathan RK, Kaminker P, Sokolova I, Bhathena A, Wang L, Naumovski L, Strickler JH (2015). Phase I study of ABT-700, an anti-c-Met antibody, in patients (pts) with advanced gastric or esophageal cancer (GEC). J Clin Oncol.

[160] Adjei AA, Schwartz B, Garmey E (2011). Early Clinical Development of ARQ 197, a Selective, Non–ATP-Competitive Inhibitor Targeting MET Tyrosine Kinase for the Treatment of Advanced Cancers. The Oncologist.

[161] Kang Y, Muro K, Ryu M, Yasui H, Nishina T, Ryoo B, Kamiya Y, Akinaga S, Boku N (2014). A phase II trial of a selective c-Met inhibitor tivantinib (ARQ 197) monotherapy as a second- or third-line therapy in the patients with metastatic gastric cancer. Invest New Drugs.

[162] Kwak EL, LoRusso P, Hamid O, Janku F, Kittaneh M, Thomas D, Chan E, Bekaii-Saab TS, Amore B, Hwang YC, Tang R, Ngarmchamnanrith G, Hong DS (2015). Clinical activity of AMG 337, an oral MET kinase inhibitor, in adult patients (pts) with MET-amplified gastroesophageal junction (GEJ), gastric (G), or esophageal (E) cancer. J Clin Oncol.

[163] Hong DS, LoRusso P, Hamid O, Beaupre DM, Janku F, Khan R, Kittaneh M, Lobert RD, Amore B, Caudillo I, Hwang YC, Tang R, Ngarmchamnanrith G, Kwak EL (2014). First-in-human study of AMG 337, a highly selective oral inhibitor of MET, in adult patients (pts) with advanced solid tumors. J Clin Oncol.

[164] Kataoka Y, Mukohara T, Tomioka H, Funakoshi Y, Kiyota N, Fujiwara Y, Yashiro M, Hirakawa K, Hirai M, Minami H (2012). Foretinib (GSK1363089), a multi-kinase inhibitor of MET and VEGFRs, inhibits growth of gastric cancer cell lines by blocking inter-receptor tyrosine kinase networks. Invest New Drugs.

[165] Qian F, Engst S, Yamaguchi K, Yu P, Won K-A, Mock L, Lou T, Tan J, Li C, Tam D, Lougheed J, Yakes FM, Bentzien F (2009). Inhibition of Tumor Cell Growth, Invasion, and Metastasis by EXEL-2880 (XL880, GSK1363089), a Novel Inhibitor of HGF and VEGF Receptor Tyrosine Kinases. Cancer Res.

[166] Shah MA, Wainberg ZA, Catenacci DVT, Hochster HS, Ford J, Kunz P, Lee F-C, Kallender H, Cecchi F, Rabe DC, Keer H, Martin A-M, Liu Y (2013). Phase II Study Evaluating 2 Dosing Schedules of Oral Foretinib (GSK1363089), cMET/VEGFR2 Inhibitor, in Patients with Metastatic Gastric Cancer. PLoS One.

[167] Yakes FM, Chen J, Tan J, Yamaguchi K, Shi Y, Yu P, Qian F, Chu F, Bentzien F, Cancilla B, Orf J, You A, Laird AD (2011). Cabozantinib (XL184), a Novel MET and VEGFR2 Inhibitor, Simultaneously Suppresses Metastasis, Angiogenesis, and Tumor Growth. Mol Cancer Ther.

[168] Wang W, Li Q, Takeuchi S, Yamada T, Koizumi H, Nakamura T, Matsumoto K, Mukaida N, Nishioka Y, Sone S, Nakagawa T, Uenaka T, Yano S (2012). Met Kinase Inhibitor E7050 Reverses Three Different Mechanisms of Hepatocyte Growth Factor–Induced Tyrosine Kinase Inhibitor Resistance in EGFR Mutant Lung Cancer. Clin Cancer Res.

[169] Molife LR, Dean EJ, Blanco-Codesido M, Krebs MG, Brunetto AT, Greystoke AP, Daniele G, Lee L, Kuznetsov G, Myint KT, Wood K, de las Heras B, Ranson MR (2014). A Phase I, Dose-Escalation Study of the Multitargeted Receptor Tyrosine Kinase Inhibitor, Golvatinib, in Patients with Advanced Solid Tumors. Clin Cancer Res.

[170] Sahai E, Marshall CJ (2002). RHO-GTPases and cancer. Nat Rev Cancer.

[171] Shang X, Marchioni F, Evelyn CR, Sipes N, Zhou X, Seibel W, Wortman M, Zheng Y (2013). Small-molecule inhibitors targeting G-protein-coupled Rho guanine nucleotide exchange factors. Proc Natl Acad Sci USA.

[172] Garnock-Jones KP (2014). Ripasudil: first global approval. Drugs.

[173] Shang X, Marchioni F, Sipes N, Evelyn CR, Jerabek-Willemsen M, Duhr S, Seibel W, Wortman M, Zheng Y (2012). Rational design of small molecule inhibitors targeting RhoA subfamily Rho GTPases. Chem Biol.

[174] Quintas-Cardama A, Verstovsek S (2013). Molecular pathways: Jak/STAT pathway: mutations, inhibitors, and resistance. Clin Cancer Res.

[175] Brooks AJ, Dai W, O'Mara ML, Abankwa D, Chhabra Y, Pelekanos RA, Gardon O, Tunny KA, Blucher KM, Morton CJ, Parker MW, Sierecki E, Gambin Y (2014). Mechanism of activation of protein kinase JAK2 by the growth hormone receptor. Science.

[176] Levine RL, Wadleigh M, Cools J, Ebert BL, Wernig G, Huntly BJ, Boggon TJ, Wlodarska I, Clark JJ, Moore S, Adelsperger J, Koo S, Lee JC (2005). Activating mutation in the tyrosine kinase JAK2 in polycythemia vera, essential thrombocythemia, and myeloid metaplasia with myelofibrosis. Cancer Cell.

[177] Verstovsek S (2009). Therapeutic potential of JAK2 inhibitors. Hematology Am Soc Hematol Educ Program.

[178] Pardanani A (2008). JAK2 inhibitor therapy in myeloproliferative disorders: rationale, preclinical studies and ongoing clinical trials. Leukemia.

[179] Plimack ER, Lorusso PM, McCoon P, Tang W, Krebs AD, Curt G, Eckhardt SG (2013). AZD1480: a phase I study of a novel JAK2 inhibitor in solid tumors. Oncologist.

[180] Qian C, Wang J, Yao J, Wang L, Xue M, Liu W, Si J (2013). Involvement of nuclear JAK2 signaling in AG490-induced apoptosis of gastric cancer cells. Anat Rec.

[181] Judd LM, Menheniott TR, Ling H, Jackson CB, Howlett M, Kalantzis A, Priebe W, Giraud AS (2014). Inhibition of the JAK2/STAT3 pathway reduces gastric cancer growth in vitro and in vivo. PLoS One.

[182] Chen T, Yang K, Yu J, Meng W, Yuan D, Bi F, Liu F, Liu J, Dai B, Chen X, Wang F, Zeng F, Xu H, Hu J, Mo X (2012). Identification and expansion of cancer stem cells in tumor tissues and peripheral blood derived from gastric adenocarcinoma patients. Cell Res.

[183] Chen W, Zhang X, Chu C, Cheung WL, Ng L, Lam S, Chow A, Lau T, Chen M, Li Y, Nie Y, Wong BC, Pang R (2013). Identification of CD44+ cancer stem cells in human gastric cancer. Hepatogastroenterology.

[184] Liu J, Ma L, Xu J, Liu C, Zhang J, Chen R, Zhou Y (2013). Spheroid body-forming cells in the human gastric cancer cell line MKN-45 possess cancer stem cell properties. Int J Oncol.

[185] Sun M, Zhou W, Zhang YY, Wang DL, Wu XL (2013). CD44 gastric cancer cells with stemness properties are chemoradioresistant and highly invasive. Oncol Lett.

[186] Xu G, Shen J, Ou Yang X, Sasahara M, Su X (2013). Cancer stem cells: the ‘heartbeat’ of gastric cancer. J Gastroenterol.

[187] Li M, Zhang B, Zhang Z, Liu X, Qi X, Zhao J, Jiang Y, Zhai H, Ji Y, Luo D (2014). Stem cell-like circulating tumor cells indicate poor prognosis in gastric cancer. Biomed Res Int.

[188] Li K, Dan Z, Nie YQ (2014). Gastric cancer stem cells in gastric carcinogenesis, progression, prevention and treatment. World J Gastroenterol.

[189] Huang J, Kalderon D (2014). Coupling of Hedgehog and Hippo pathways promotes stem cell maintenance by stimulating proliferation. J Cell Biol.

[190] Stojnev S, Krstic M, Ristic-Petrovic A, Stefanovic V, Hattori T (2014). Gastric cancer stem cells: therapeutic targets. Gastric Cancer.

[191] Kang DH, Han ME, Song MH, Lee YS, Kim EH, Kim HJ, Kim GH, Kim DH, Yoon S, Baek SY, Kim BS, Kim JB, Oh SO (2009). The role of hedgehog signaling during gastric regeneration. J Gastroenterol.

[192] Song Z, Yue W, Wei B, Wang N, Li T, Guan L, Shi S, Zeng Q, Pei X, Chen L (2011). Sonic hedgehog pathway is essential for maintenance of cancer stem-like cells in human gastric cancer. PLoS One.

[193] Tanaka T, Arai M, Minemura S, Oyamada A, Saito K, Jiang X, Tsuboi M, Sazuka S, Maruoka D, Matsumura T, Nakagawa T, Sugaya S, Kanda T (2014). Expression level of sonic hedgehog correlated with the speed of gastric mucosa regeneration in artificial gastric ulcers. J Gastroenterol Hepatol.

[194] Gupta S, Takebe N, Lorusso P (2010). Targeting the Hedgehog pathway in cancer. Ther Adv Med Oncol.

[195] Lee SY, Han HS, Lee KY, Hwang TS, Kim JH, Sung IK, Park HS, Jin CJ, Choi KW (2007). Sonic hedgehog expression in gastric cancer and gastric adenoma. Oncol Rep.

[196] Sherman AE, Zavros Y (2011). Role of Sonic Hedgehog signaling during progression from inflammation to cancer in the stomach. World J Gastrointest Pathophysiol.

[197] Kim JH, Choi YJ, Lee SH, Shin HS, Lee IO, Kim YJ, Kim H, Yang WI, Kim H, Lee YC (2010). Effect of Helicobacter pylori infection on the sonic hedgehog signaling pathway in gastric cancer cells. Oncol Rep.

[198] Kim JH, Shin HS, Lee SH, Lee I, Lee YS, Park JC, Kim YJ, Chung JB, Lee YC (2010). Contrasting activity of Hedgehog and Wnt pathways according to gastric cancer cell differentiation: relevance of crosstalk mechanisms. Cancer Sci.

[199] Yanai K, Nakamura M, Akiyoshi T, Nagai S, Wada J, Koga K, Noshiro H, Nagai E, Tsuneyoshi M, Tanaka M, Katano M (2008). Crosstalk of hedgehog and Wnt pathways in gastric cancer. Cancer Lett.

[200] Seto M, Ohta M, Asaoka Y, Ikenoue T, Tada M, Miyabayashi K, Mohri D, Tanaka Y, Ijichi H, Tateishi K, Kanai F, Kawabe T, Omata M (2009). Regulation of the hedgehog signaling by the mitogen-activated protein kinase cascade in gastric cancer. Mol Carcinog.

[201] Kim JY, Ko GH, Lee YJ, Ha WS, Choi SK, Jung EJ, Jeong CY, Ju YT, Jeong SH, Hong SC (2012). Prognostic value of sonic hedgehog protein expression in gastric cancer. Jpn J Clin Oncol.

[202] Yoo YA, Kang MH, Lee HJ, Kim BH, Park JK, Kim HK, Kim JS, Oh SC (2011). Sonic hedgehog pathway promotes metastasis and lymphangiogenesis via activation of Akt, EMT, and MMP-9 pathway in gastric cancer. Cancer Res.

[203] Saze Z, Terashima M, Kogure M, Ohsuka F, Suzuki H, Gotoh M (2012). Activation of the sonic hedgehog pathway and its prognostic impact in patients with gastric cancer. Dig Surg.

[204] Niu Y, Li F, Tang B, Shi Y, Hao Y, Yu P (2014). Clinicopathological correlation and prognostic significance of sonic hedgehog protein overexpression in human gastric cancer. Int J Clin Exp Pathol.

[205] Yan R, Peng X, Yuan X, Huang D, Chen J, Lu Q, Lv N, Luo S (2013). Suppression of growth and migration by blocking the Hedgehog signaling pathway in gastric cancer cells. Cell Oncol (Dordr).

[206] Bai R, Zhao H, Zhang X, Du S (2014). Characterization of sonic hedgehog inhibition in gastric carcinoma cells. Oncol Lett.

[207] Wan J, Zhou J, Zhao H, Wang M, Wei Z, Gao H, Wang Y, Cui H (2014). Sonic hedgehog pathway contributes to gastric cancer cell growth and proliferation. Biores Open Access.

[208] Yoon C, Park do J, Schmidt B, Thomas NJ, Lee HJ, Kim TS, Janjigian YY, Cohen DJ, Yoon SS (2014). CD44 expression denotes a subpopulation of gastric cancer cells in which Hedgehog signaling promotes chemotherapy resistance. Clin Cancer Res.

[209] Sobanko JF, Okman J, Miller C (2013). Vismodegib: a hedgehog pathway inhibitor for locally advanced and metastatic basal cell carcinomas. J Drugs Dermatol.

[210] Gajjar A, Stewart CF, Ellison DW, Kaste S, Kun LE, Packer RJ, Goldman S, Chintagumpala M, Wallace D, Takebe N, Boyett JM, Gilbertson RJ, Curran T (2013). Phase I study of vismodegib in children with recurrent or refractory medulloblastoma: a pediatric brain tumor consortium study. Clin Cancer Res.

[211] Cohen DJ, Christos PJ, Sparano JA, Kindler HL, Catenacci D, Bekaii-Saab TB, Tahiri S, Janjigian YY, Gibson MK, Chan E, Rajdev L, Urba S, Wade JL (2013). A randomized phase II study of vismodegib (V), a hedgehog (HH) pathway inhibitor, combined with FOLFOX in patients (pts) with advanced gastric and gastroesophageal junction (GEJ) carcinoma: A New York Cancer Consortium led study. J Clin Oncol.

[212] Berlin J, Bendell JC, Hart LL, Firdaus I, Gore I, Hermann RC, Mulcahy MF, Zalupski MM, Mackey HM, Yauch RL, Graham RA, Bray GL, Low JA (2013). A randomized phase II trial of vismodegib versus placebo with FOLFOX or FOLFIRI and bevacizumab in patients with previously untreated metastatic colorectal cancer. Clin Cancer Res.

[213] Brechbiel J, Miller-Moslin K, Adjei AA (2014). Crosstalk between hedgehog and other signaling pathways as a basis for combination therapies in cancer. Cancer Treat Rev.

[214] Schepers A, Clevers H (2012). Wnt signaling, stem cells, and cancer of the gastrointestinal tract. Cold Spring Harb Perspect Biol.

[215] Takahashi-Yanaga F, Kahn M (2010). Targeting Wnt signaling: can we safely eradicate cancer stem cells?. Clin Cancer Res.

[216] Anastas JN, Moon RT (2013). WNT signalling pathways as therapeutic targets in cancer. Nat Rev Cancer.

[217] Ooi CH, Ivanova T, Wu J, Lee M, Tan IB, Tao J, Ward L, Koo JH, Gopalakrishnan V, Zhu Y, Cheng LL, Lee J, Rha SY (2009). Oncogenic pathway combinations predict clinical prognosis in gastric cancer. PLoS Genet.

[218] Mao J, Fan S, Ma W, Fan P, Wang B, Zhang J, Wang H, Tang B, Zhang Q, Yu X, Wang L, Song B, Li L (2014). Roles of Wnt/beta-catenin signaling in the gastric cancer stem cells proliferation and salinomycin treatment. Cell Death Dis.

[219] Bizama C, Benavente F, Salvatierra E, Gutierrez-Moraga A, Espinoza JA, Fernandez EA, Roa I, Mazzolini G, Sagredo EA, Gidekel M, Podhajcer OL (2014). The low-abundance transcriptome reveals novel biomarkers, specific intracellular pathways and targetable genes associated with advanced gastric cancer. Int J Cancer.

[220] Curtin JC, Lorenzi MV (2010). Drug discovery approaches to target Wnt signaling in cancer stem cells. Oncotarget.

[221] Lenz HJ, Kahn M (2014). Safely targeting cancer stem cells via selective catenin coactivator antagonism. Cancer Sci.

[222] Holland JD, Klaus A, Garratt AN, Birchmeier W (2013). Wnt signaling in stem and cancer stem cells. Curr Opin Cell Biol.

[223] Singh SR (2013). Gastric cancer stem cells: a novel therapeutic target. Cancer Lett.

[224] Takaishi S, Okumura T, Tu S, Wang SS, Shibata W, Vigneshwaran R, Gordon SA, Shimada Y, Wang TC (2009). Identification of gastric cancer stem cells using the cell surface marker CD44. Stem Cells.

[225] Zhang C, Li C, He F, Cai Y, Yang H (2011). Identification of CD44+CD24+ gastric cancer stem cells. J Cancer Res Clin Oncol.

[226] Yang L, Ping YF, Yu X, Qian F, Guo ZJ, Qian C, Cui YH, Bian XW (2011). Gastric cancer stem-like cells possess higher capability of invasion and metastasis in association with a mesenchymal transition phenotype. Cancer Lett.

[227] Han ME, Jeon TY, Hwang SH, Lee YS, Kim HJ, Shim HE, Yoon S, Baek SY, Kim BS, Kang CD, Oh SO (2011). Cancer spheres from gastric cancer patients provide an ideal model system for cancer stem cell research. Cell Mol Life Sci.

[228] de Sousa EM, Vermeulen L, Richel D, Medema JP (2011). Targeting Wnt signaling in colon cancer stem cells. Clin Cancer Res.

[229] Vermeulen L, De Sousa EMF, van der Heijden M, Cameron K, de Jong JH, Borovski T, Tuynman JB, Todaro M, Merz C, Rodermond H, Sprick MR, Kemper K, Richel DJ, Stassi G, Medema JP (2010). Wnt activity defines colon cancer stem cells and is regulated by the microenvironment. Nat Cell Biol.

[230] Lamb R, Ablett MP, Spence K, Landberg G, Sims AH, Clarke RB (2013). Wnt pathway activity in breast cancer sub-types and stem-like cells. PLoS One.

[231] Kwon MJ, Shin YK (2013). Regulation of ovarian cancer stem cells or tumor-initiating cells. Int J Mol Sci.

[232] Cui J, Jiang W, Wang S, Wang L, Xie K (2012). Role of Wnt/beta-catenin signaling in drug resistance of pancreatic cancer. Curr Pharm Des.

[233] Teng Y, Wang X, Wang Y, Ma D (2010). Wnt/beta-catenin signaling regulates cancer stem cells in lung cancer A549 cells. Biochem Biophys Res Commun.

[234] Koch U, Radtke F (2007). Notch and cancer: a double-edged sword. Cell Mol Life Sci.

[235] Kopan R IM (2009). The canonical Notch signaling pathway: unfolding the activation mechanism. Cell.

[236] Leong KG, Karsan A (2005). Recent insights into the role of Notch signaling in tumorigenesis. Blood.

[237] Kang H, An H, Song J, Kim T, Heo J, Ahn D, Kim G (2012). Notch3 and Jagged2 contribute to gastric cancer development and to glandular differentiation associated with MUC2 and MUC5AC expression. Histopathology.

[238] Brzozowa M, Mielańczyk L, Michalski M, Malinowski L, Kowalczyk-Ziomek G, Helewski K, Harabin-Słowińska M, Wojnicz R (2013). Role of Notch signaling pathway in gastric cancer pathogenesis. Contemp Oncol (Pozn).

[239] Du X, Cheng Z, Wang YH, Guo ZH, Zhang SQ, Hu JK, Zhou ZG (2014). Role of Notch signaling pathway in gastric cancer: a meta-analysis of the literature. World J Gastroenterol.

[240] Yeh T, Wu C, Hsu K, Liao W, Yang M, Li AF, Wang A, Kuo M, Chi C (2009). The activated Notch1 signal pathway is associated with gastric cancer progression through cyclooxygenase-2. Cancer Res.

[241] Lee SH, Jeong EG, Yoo NJ, Lee SH (2007). Mutational analysis of NOTCH1, 2, 3 and 4 genes in common solid cancers and acute leukemias. APMIS.

[242] Miele L (2006). Notch signaling. Clin Cancer Res.

[243] Rizzo P, Osipo C, Foreman K, Golde T, Osborne B, Miele L (2008). Rational targeting of Notch signaling in cancer. Oncogene.

[244] Wang Z, Li Y, Ahmad A, Azmi AS, Banerjee S, Kong D, Sarkar FH (2010). Targeting Notch signaling pathway to overcome drug resistance for cancer therapy. Biochim Biophys Acta.

[245] Shih L, Wang T (2007). Notch Signaling, γ-Secretase Inhibitors, and Cancer Therapy. Cancer Res.

[246] Johnson R, Halder G (2014). The two faces of Hippo: targeting the Hippo pathway for regenerative medicine and cancer treatment. Nat Rev Drug Discov.

[247] Lam-Himlin DM, Daniels JA, Gayyed MF, Dong J, Maitra A, Pan D, Montgomery EA, Anders RA (2006). The hippo pathway in human upper gastrointestinal dysplasia and carcinoma: a novel oncogenic pathway. Int J Gastrointest Cancer.

[248] Zhang J, Yang YC, Zhu JS, Zhou Z, Chen WX (2012). Clinicopathologic characteristics of YES-associated protein 1 overexpression and its relationship to tumor biomarkers in gastric cancer. Int J Immunopathol Pharmacol.

[249] Song M, Cheong JH, Kim H, Noh SH, Kim H (2012). Nuclear expression of Yes-associated protein 1 correlates with poor prognosis in intestinal type gastric cancer. Anticancer Res.

[250] Xu ZP, Zhu JS, Zhang Q, Wang XY (2011). A breakdown of the Hippo pathway in gastric cancer. Hepatogastroenterology.

[251] Liu-Chittenden Y, Huang B, Shim JS, Chen Q, Lee SJ, Anders RA, Liu JO, Pan D (2012). Genetic and pharmacological disruption of the TEAD-YAP complex suppresses the oncogenic activity of YAP. Genes Dev.

[252] Park HW, Guan KL (2013). Regulation of the Hippo pathway and implications for anticancer drug development. Trends Pharmacol Sci.

[253] Huggett MT, Jermyn M, Gillams A, Illing R, Mosse S, Novelli M, Kent E, Bown SG, Hasan T, Pogue BW, Pereira SP (2014). Phase I/II study of verteporfin photodynamic therapy in locally advanced pancreatic cancer. Br J Cancer.

[254] Waddell T, Chau I, Cunningham D, Gonzalez D, Okines AF, Okines C, Wotherspoon A, Saffery C, Middleton G, Wadsley J, Ferry D, Mansoor W, Crosby T (2013). Epirubicin, oxaliplatin, and capecitabine with or without panitumumab for patients with previously untreated advanced oesophagogastric cancer (REAL3): a randomised, open-label phase 3 trial. Lancet Oncol.

[255] Barros-Silva JD, Leitao D, Afonso L, Vieira J, Dinis-Ribeiro M, Fragoso M, Bento MJ, Santos L, Ferreira P, Rego S, Brandao C, Carneiro F, Lopes C, Schmitt F, Teixeira MR (2009). Association of ERBB2 gene status with histopathological parameters and disease-specific survival in gastric carcinoma patients. Br J Cancer.

[256] Yano T, Doi T, Ohtsu A, Boku N, Hashizume K, Nakanishi M, Ochiai A (2006). Comparison of HER2 gene amplification assessed by fluorescence in situ hybridization and HER2 protein expression assessed by immunohistochemistry in gastric cancer. Oncol Rep.

[257] Halon A, Donizy P, Biecek P, Rudno-Rudzinska J, Kielan W, Matkowski R (2012). HER-2 expression in immunohistochemistry has no prognostic significance in gastric cancer patients. Scientific World Journal.

[258] Xiao L, Wang Y, Li W, Du Y (2009). The role of mTOR and phospho-p70S6K in pathogenesis and progression of gastric carcinomas: an immunohistochemical study on tissue microarray. J Exp Clin Canc Res.

[259] Ma X, Chen K, Huang S, Zhang X, Adegboyega PA, Evers BM, Zhang H, Xie J (2005). Frequent activation of the hedgehog pathway in advanced gastric adenocarcinomas. Carcinogenesis.

